# Progression to Loss of Ambulation Among Patients with Autosomal Recessive Limb-girdle Muscular Dystrophy: A Systematic Review

**DOI:** 10.3233/JND-210771

**Published:** 2022-07-01

**Authors:** Ivana F. Audhya, Antoinette Cheung, Shelagh M. Szabo, Emma Flint, Conrad C. Weihl, Katherine L. Gooch

**Affiliations:** a Sarepta Therapeutics Inc, Cambridge MA, USA; b Broadstreet HEOR, Vancouver, BC, V6A 1A4 Canada; c Washington University School of Medicine, St. Louis, MO, USA

**Keywords:** Muscular dystrophies, limb-girdle, age of onset, disease progression, walking, systematic review

## Abstract

**Background:**

The impact of age at autosomal recessive limb girdle muscular dystrophy (LGMDR) onset on progression to loss of ambulation (LOA) has not been well established, particularly by subtype.

**Objectives:**

To describe the characteristics of patients with adult-, late childhood-, and early childhood-onset LGMDR by subtype and characterize the frequency and timing of LOA.

**Methods:**

A systematic review was conducted in MEDLINE, Embase and the Cochrane library. Frequency and timing of LOA in patients with LGMDR1, LGMDR2/Miyoshi myopathy (MM), LGMDR3-6, LGMDR9, and LGMDR12 were synthesized from published data.

**Results:**

In 195 studies, 695 (43.4%) patients had adult-, 532 (33.2%) had late childhood-, and 376 (23.5%) had early childhood-onset of disease across subtypes among those with a reported age at onset (*n* = 1,603); distribution of age at onset varied between subtypes. Among patients with LOA (*n* = 228), adult-onset disease was uncommon in LGMDR3-6 (14%) and frequent in LGMDR2/MM (42%); LGMDR3-6 cases with LOA primarily had early childhood-onset (74%). Mean (standard deviation [SD]) time to LOA varied between subtypes and was shortest for patients with early childhood-onset LGMDR9 (12.0 [4.9] years, *n* = 19) and LGMDR3-6 (12.3 [10.7], *n* = 56) and longest for those with late childhood-onset LGMDR2/MM (21.4 [11.5], *n* = 36).

**Conclusions:**

This review illustrated that patients with early childhood-onset disease tend to have faster progression to LOA than those with late childhood- or adult-onset disease, particularly in LGMDR9. These findings provide a greater understanding of progression to LOA by LGMDR subtype, which may help inform clinical trial design and provide a basis for natural history studies.

## INTRODUCTION

Limb girdle muscular dystrophy (LGMD) comprises a group of rare muscular dystrophies caused by mutations in genes encoding proteins involved with muscle maintenance, function, and repair [1]. Over 30 subtypes of LGMD have been identified, of which 90% are autosomal recessive (LGMDR) [2, 3]. The most common of these (along with their European NeuroMuscular Centre [ENMC] nomenclature/original nomenclature, and affected protein), are calpainopathy (LGMDR1/LGMD2A, calpain-3), dysferlinopathy (LGMDR2/LGMD2B and Miyoshi myopathy [MM], dysferlin), sarcoglycanopathies (LGMDR3/LGMD2D, α-sarcoglycan; LGMDR4/LGMD2E, β-sarcoglycan; LGMDR5/LGMD2 C, γ-sarcoglycan; LGMDR6/LGMD2F, *δ*-sarcoglycan), fukutin-related protein (FKRP)-related dystroglycanopathy (LGMDR9/LGMD2I, FKRP), and anoctaminopathy (LGMDR12/LGMD2 L, anoctamin-5).

Data on the natural history of LGMDR are few and studies describing larger clinical cohorts followed over long periods of time are lacking. Nonetheless, understanding the clinical course and likely disease prognosis is important. Patients with LGMD, including LGMDR, primarily present with proximal skeletal muscle weakness [4], but the clinical course of different LGMDR subtypes appears highly variable and the age at disease onset can range from childhood through adulthood. Disease severity also varies from mild forms in which patients have relatively normal function, to severe forms with rapid onset and progression [1, 4]. Patients with LGMD may experience signs and symptoms of muscle weakness at any age and these typically worsen over time [3, 5]. At an early stage, patients may present with an abnormal walking gait, difficulties in walking, and/or difficulties in running. As the disease progresses, ambulatory function may deteriorate, patients may require walking assistance, and ultimately progress to loss of ambulation (LOA) and wheelchair dependence.

Progression to LOA has been described across LGMDR subtypes [6, 7]; however, it is unclear how the frequency and timing of LOA compares between patients with different subtypes. Previous studies have suggested that the clinical trajectories of patients with LGMD onset before 10 years of age (i.e. early childhood onset) may differ from those with onset after 10 years of age [8]. Although there has been some indication that patients with pediatric-onset LGMDR may experience a more severe progression than those with adult-onset disease [9], the impact of age at symptomatic disease onset on progression to LOA has not been well established. Understanding the natural history by subtype and age at onset is important to characterize disease burden and understand likely disease trajectories among those with LGMDR. In the absence of data from large clinical studies, a systematic review can help fill this gap. As such, through a systematic review and synthesis of the published literature, this study sought to describe the characteristics of patients with pediatric- and adult-onset LGMDR according to subtype and to characterize the frequency and timing of LOA among patients with pediatric- and adult-onset LGMDR, by subtype.

## METHODS

### Search strategy and study identification

A systematic literature review was conducted in MEDLINE, Embase and the Cochrane library to identify all published data from database inception to September 3, 2019, on the frequency and timing of LOA in patients with LGMDR1, LGMDR2/MM, LGMDR3-6, LGMDR9, and LGMDR12. The search strategy was developed to include terms related to the Population, Exposure/Comparator, Outcomes, Study design (PECOS) criteria specified to be of interest for the study (Supplementary Table 1). Two reviewers worked independently to screen all abstracts identified by the search strategy against the PECOS criteria, and then reviewed the full text of all potentially relevant abstracts. If any discrepancies occurred between the studies selected for inclusion by the two reviewers, a third reviewer provided arbitration. Patient-level data from epidemiologic studies (retrospective and prospective cohort studies), clinical trials, as well as case series and reports were included.

### Outcomes and data synthesis

Pediatric-onset disease was characterized based on two categories: onset before age 10 (early childhood-onset) and onset from age 10 to 18 years (late childhood-onset). Adult-onset was defined as disease onset at 18 years or older. Patients whose age at LGMD onset was reported descriptively rather than numerically were analyzed according to the category corresponding to the appropriate age at onset definition. For example, those whose age at onset was reported as occurring “in their third decade” were categorized as having adult-onset disease. Baseline demographics and clinical characteristics of the patients included in the studies were characterized, using counts and proportions, and means and standard deviations (SD), as appropriate. These were stratified by age at onset and LGMDR subtype. Patients were considered to have LOA if they were described as being wheelchair dependent or non-ambulant. Those who were described as ambulatory at their last assessment were considered to not have LOA.

The total number of patients with patient-level data in the included studies and the number of patients with age at LGMD onset reported (and the subset among whom an ambulatory status was reported) were determined by subtype and among the overall sample in order to characterize data availability.

### Outcomes of interest (overall and for each LGMDR subtype)

The following outcomes were extracted from the overall sample and for each LGMDR subtype. Among patients with age at LGMD onset reported, the n (%) with adult- (>18 years), late childhood- (10-18 years), and early childhood-onset (<10 years) LGMDR was tabulated. This was determined for the overall cohort as well as separately for male and female patients. Among patients with adult-, late childhood-, and early childhood-onset of disease, the n (%) with ambulatory status reported was assessed; patients could be either ambulatory or non-ambulatory at the time of each assessment. Among those with LOA, mean (SD) time from LGMD onset to LOA, stratified by age of onset (adult-, late childhood-, and early childhood-onset LGMDR, as per the above) was tabulated. Time to LOA was calculated for patients whose age at disease onset was reported numerically, rather than descriptively (e.g., in the third decade). Patients were analyzed based on the subgroup reported by study authors, except for LGMDR2 and MM which were combined into an overall dysferlinopathies subgroup for analysis (LGMDR2/MM).

As all data used in the study were from previously published sources, participant consent or ethical review and approval was not necessary.

## RESULTS

### Study characteristics

**Fig. 1:**
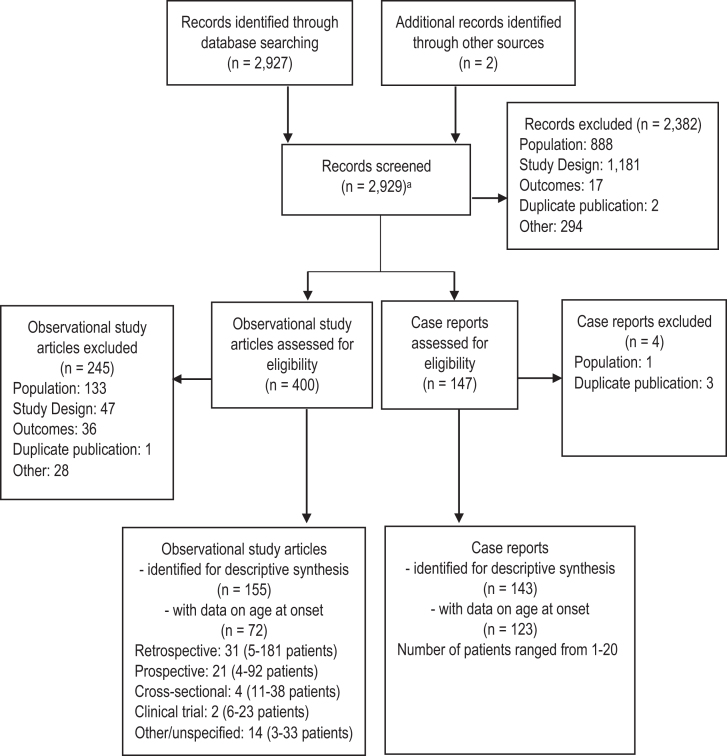
PRISMA diagram. ^a^Removal of duplicates was built into the search strategy.

The systematic search identified 2,929 abstracts which after screening resulted in the inclusion of 298 studies, of which 195 studies provided data informing this analysis (Fig. 1). Data were primarily derived from case reports and case series (*n* = 123) [10–132], although patient-level data were also available from 31 retrospective [133–163], 21 prospective [164–184], and 4 cross-sectional studies [185–188], as well as 2 randomized controlled trials [189, 190] and 14 reports based on other designs (Supplementary Table 2) [98, 191–203]. Of studies reporting geographic location, over 35 countries were represented, with the most studies conducted in the United States (*n* = 28) and Italy (*n* = 23).

### Data availability

**Fig. 2:**
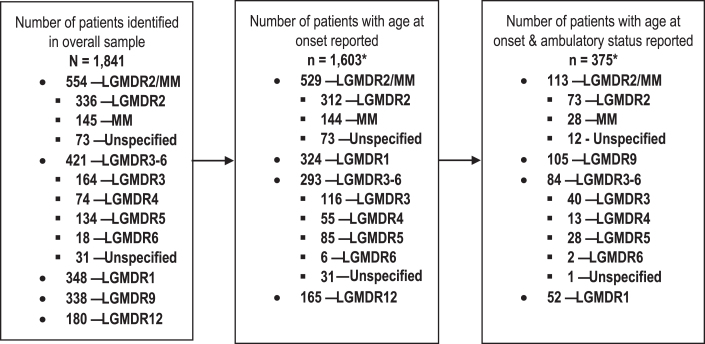
Summary of data availability across LGMDR subtypes. *Includes patients whose age at onset was reported descriptively rather than numerically, with sufficient information to be categorized as having either adult-, late childhood-, or early childhood-onset disease (e.g. onset in “first decade”). **Abbreviations:** LGMDR, autosomal recessive limb girdle muscular dystrophy; MM, Miyoshi myopathy.

In total, data from 1,841 patients were identified to comprise the overall sample (Fig. 2); of these, 1,603 (87.1%) patients had a reported age at onset. LGMDR2/MM and LGMDR1 were the most common subtypes among patients with a reported age at onset (*n* = 529, 33.0%; *n* = 324, 20.2%, respectively), while LGMDR6 was uncommon (*n* = 6, 0.4%). Among those with age at onset reported, 375 patients also had ambulatory status reported (23.4%), and the percentages with ambulatory status reported ranged from 0.5% (*n* = 2; LGMDR6) to 30.1% (*n* = 113; LGMDR2/MM) when considered by subtype. Data on ambulatory status were generally cross-sectional in nature; ambulatory function was typically reported at one assessment only.

### Patient characteristics

#### Patient characteristics by age at onset category

**Fig. 3:**
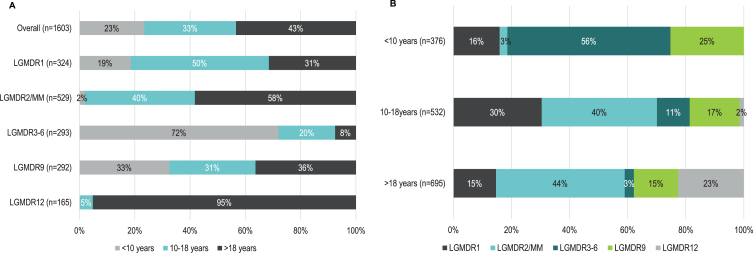
A) Distribution of early childhood-, late childhood- and adult- onset LGMD, by subtype; B) distribution of LGMD subtypes by age at onset category. **Note:** Percentages are rounded to the nearest whole number. **Abbreviations:** LGMDR, autosomal recessive limb girdle muscular dystrophy; MM, Miyoshi myopathy.

Of the 1,603 patients with a reported age at onset, 695 (43.4%) had adult-, 532 (33.2%) had late childhood-, and 376 (23.5%) had early childhood-onset disease overall (Fig. 3A). The distribution of patients with pediatric- compared to adult-onset disease was highly variable between subtypes. Patients with LGMDR12 had onset predominantly in adulthood (95.2%), while adult-onset disease was infrequent among patients with LGMDR3-6 (7.5%). When the distribution of pediatric- compared to adult-onset disease was evaluated by sex, there were no real differences by sex among included patients with age at onset data, although the number of female patients with LGMDR12 (*n* = 39) was comparatively lower than the number of male patients with LGMDR12 (*n* = 124) identified for this analysis (data not shown). Among patients with adult-onset (*n* = 695) and late childhood-onset LGMDR (*n* = 532), the most common subtype was LGMDR2/MM (*n* = 308, 44.3% adult-onset; *n* = 211, 39.7% late childhood-onset; Table 1, Fig. 3B). Among those with early childhood-onset disease (*n* = 376), LGMDR3-6 was the most common subtype (*n* = 211; 56.1%). There were no patients with early childhood-onset LGMDR12.

**Table 1:** Characteristics of patients contributing data to the analysis, stratified by age at onset and subtype (n = 1,603)

Subtype	N with age at onset reported	Mean (SD) age at onset	n with sex reported	n male (%)
**Adult-onset (>18 years)**
Overall*	**695**	**29.2 (10.1)**	**672**	**359 (51.7)**
**LGMDR1**	102	30.4 (10.4)	100	39 (38.2)
**LGMDR2/MM**	308	26.3 (7.9)	290	140 (45.5)
**LGMDR3-6**	22	29.9 (8.2)	21	9 (40.9)
**LGMDR5**	7	23	7	3 (42.9)
**LGMDR3**	12	32.1 (9.5)	11	4 (33.3)
**LGMDR4**	0	N/A	0	0
**LGMDR6**	0	N/A	0	0
**LGMDR9**	106	29.6 (8.2)	106	54 (50.9)
**LGMDR12**	157	35.8 (13.1)	155	117 (74.5)
**Late childhood-onset (10-18 years)**
Overall*	**532**	**14.0 (2.7)**	**516**	**242 (45.5)**
**LGMDR1**	162	13.1 (2.5)	160	62 (38.3)
**LGMDR2/MM**	211	15.5 (2.1)	197	97 (46.0)
**LGMDR3-6**	60	11.8 (2.1)	60	31 (51.7)
**LGMDR5**	10	12.3 (2.8)	10	5 (50.0)
**LGMDR3**	32	11.6 (1.7)	32	14 (43.8)
**LGMDR4**	11	12.8 (2.4)	11	8 (72.7)
**LGMDR6**	0	N/A	0	0
**LGMDR9**	91	13.7 (2.7)	91	45 (49.5)
**LGMDR12**	8	15.2 (2.3)	8	7 (87.5)
**Early childhood-onset (<10 years)**
Overall*	**376**	**5.1 (2.5)**	**374**	**201 (53.5)**
**LGMDR1**	60	5.9 (2.4)	60	24 (40.0)
**LGMDR2/MM**	10	4.3 (2.6)	8	8 (80.0)
**LGMDR3-6**	211	5.1 (2.3)	211	112 (53.1)
**LGMDR5**	68	5.5 (2.3)	68	39 (57.4)
**LGMDR3**	72	5.1 (2.4)	72	35 (48.6)
**LGMDR4**	44	4.4 (2.4)	44	23 (52.3)
**LGMDR6**	6	3.8 (2.3)	6	4 (66.7)
**LGMDR9**	95	4.8 (2.8)	95	57 (60.0)
LGMDR12	0	N/A	0	0

*Within each age at onset category, “Overall” refers to unique patients in that category. As patients with LGMDR3, LGMDR4, LGMDR5, and LGMDR6 may be counted in both the individual subtype and combined LGMDR3-6 groups, the number of patients added across subtypes exceed the counts reflected under “Overall.” **Abbreviations:** LGMDR, autosomal recessive limb girdle muscular dystrophy; MM, Miyoshi myopathy; N/A, not applicable; SD, standard deviation.

### Age at onset and ambulatory status

The frequency of ambulation assessment varied across age at onset categories and LGMDR subtypes (Table 2). Patients with early childhood-onset disease had ambulation assessed most often (33.2%, 121/376), while ambulation was infrequently assessed in patients with adult-onset disease (17.6%, 122/695). Among the different subtypes, ambulation assessments were generally common in LGMDR3-6 and LGMDR9 regardless of age at onset. The frequency of ambulation assessment was lowest in LGMDR1 (11.8% of adult-onset, 18.5% of late childhood-onset, and 16.7% of early childhood-onset disease).

**Table 2:** Ambulatory status and age at ambulation assessment, stratified by age at onset and subtype

Subtype	n (%) with ambulation assessment	n (%) LOA	Mean (SD) age at LOA	Mean (SD) age at last assessment if ambulatory
**Adult-onset (>18 years)**
**Overall (*n*** = **695)**	**122 (17.6)**	**64 (52.5)**	**44.7 (13.6)**	**43.4 (12.0)**
**LGMDR1 (*n*** = **102)**	12 (11.8)	11 (91.7)	48.4 (14.2)	43.0
**LGMDR2/MM (*n*** = **308)**	56 (18.2)	32 (57.1)	45.2 (13.1)	37.5 (10.6)
**LGMDR3-6 (*n*** = **22)**	11 (50.0)	11 (100.0)	32.5 (6.9)	N/A
**LGMDR9 (*n*** = **106)**	23 (21.7)	5 (21.7)	49.4 (14.4)	43.2 (12.4)
**LGMDR12 (*n*** = **157)**	20 (12.7)	5 (25.0)	55.0 (12.5)	53.2 (7.3)
**Late childhood-onset (10-18 years)**
**Overall (*n*** = **532)**	**132 (24.8)**	**78 (59.1)**	**32.8 (11.4)**	**26.5 (11.4)**
**LGMDR1 (*n*** = **162)**	30 (18.5)	17 (56.7)	30.6 (12.1)	20.9 (7.8)
**LGMDR2/MM (*n*** = **211)**	54 (25.6)	42 (77.8)	36.2 (11.1)	24.0 (6.6)
**LGMDR3-6 (*n*** = **60)**	13 (21.7)	9 (69.2)	21.7 (9.4)	28.3 (14.3)
**LGMDR9 (*n*** = **91)**	34 (37.4)	10 (29.4)	32.1 (8.6)	30.2 (13.4)
**LGMDR12 (*n*** = **8)**	1 (12.5)	0 (0.0)	N/A	35.0
**Early childhood-onset (<10 years)**
**Overall (*n*** = **376)**	**121 (32.2)**	**86 (71.1)**	**17.7 (10.6)**	**13.1 (10.1)**
**LGMDR1 (*n*** = **60)**	10 (16.7)	7 (70.0)	21.6 (10.6)	17.2 (7.1)
**LGMDR2/MM (*n*** = **10)**	3 (30.0)	3 (100.0)	36.0 (12.1)	N/A
**LGMDR3-6 (*n*** = **211)**	60 (28.4)	57 (95.0)	16.9 (11.1)	8.0 (1.0)
**LGMDR9 (*n*** = **95)**	48 (50.5)	19 (39.6)	15.5 (5.9)	13.2 (10.8)
**LGMDR12 (*n*** = **0)**	N/A	N/A	N/A	N/A

**Abbreviations:** LGMDR, autosomal recessive limb girdle muscular dystrophy; LOA, loss of ambulation; MM, Miyoshi myopathy; N/A, not applicable; SD, standard deviation.

LOA was observed in 60.8% (228/375) of patients with ambulatory function reported. Among patients with adult-onset disease, LOA was reported in 52.5%, and at a mean (SD) age of 44.7 (13.6) years (Table 2). LOA was most frequently reported among patients with LGMDR3-6, who also experienced the earliest LOA (mean [SD]: 32.5 [6.9] years) in this age at onset group. Among patients with late childhood-onset disease, 59.1% experienced LOA overall, at a mean (SD) age of 32.8 (11.4) years (Table 2). Compared to patients with adult-onset disease (52.5%), the percentage of patients with late childhood-onset disease who had LOA was slightly higher. Among patients with early childhood-onset disease, 71.1% experienced LOA overall, at a mean (SD) age of 17.7 (10.6) years (Table 2). LOA was most common in this age at onset group. This trend was generally consistent across subtypes, except in cases where low numbers of patients with ambulatory status were available. The number and percentage of patients with LOA and adult- or late childhood-onset disease was highest for LGMDR2/MM (*n* = 32, 57.1%; *n* = 42, 77.8%, respectively); while n (%) with LOA and early childhood-onset was highest for LGMDR3-6 (*n* = 57, 95.0%). All 3 patients with early childhood-onset LGMDR2/MM with an ambulatory assessment had LOA and their mean age at LOA was comparable to the mean age of LOA for the late-childhood onset patients.

### Time to LOA

**Fig. 4:**
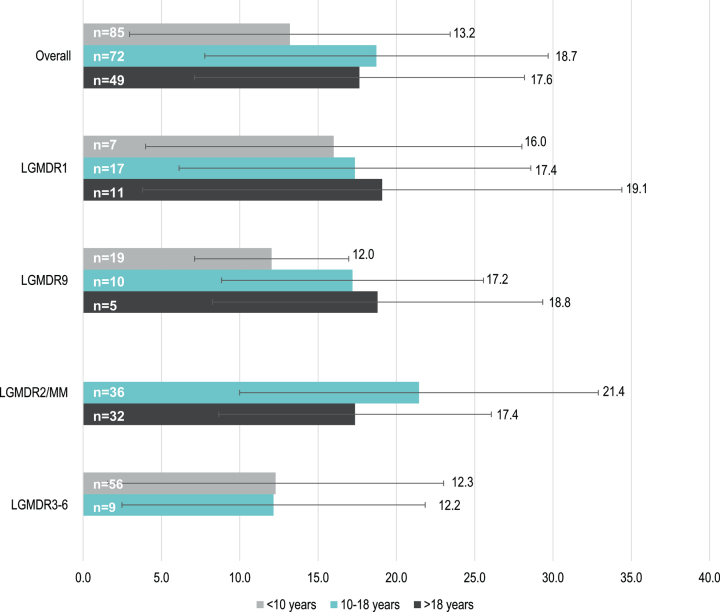
Mean (SD) time (years) to LOA of early childhood-, late childhood- and adult- onset LGMDR, by subtype. **Note: time to LOA was only determined for patients whose age at onset was reported numerically; e.g. patients who were reported as having onset “in adulthood” are not reflected. In addition, subtypes and age groups with n<5 are not reflected (e.g. LGMDR12). **Abbreviations:** LGMDR, autosomal recessive limb girdle muscular dystrophy; LOA, loss of ambulation; MM, Miyoshi myopathy; SD, standard deviation.

Data to assess time from disease onset to LOA according to age at onset category were limited. Mean (SD) time to LOA varied between subtypes and was shortest for patients with early childhood-onset LGMDR9 (12.0 [4.9] years, *n* = 19) and LGMDR3-6 (12.3 [10.7] years, *n* = 56) and longest for those with late childhood-onset LGMDR2/MM (21.4 [11.5] years, *n* = 36) (Fig. 4). In LGMDR1 and LGMDR9, patients with earlier disease onset appeared to have a faster progression to LOA than those with later disease onset.

## DISCUSSION

This systematic review described the frequency and timing of LOA in LGMDR according to age at disease onset and subtype. LOA represents a severe manifestation of LGMDR, in which muscle weakness and contractures have progressed and worsened over time [204]. Given the heterogeneity of clinical progression among different patients [6–8], the occurrence of LOA was characterized separately for each LGMDR subtype, and for patients with adult-, late childhood-, and early childhood-onset disease.

Understanding the natural history of the progression to LOA and intervening prior to its occurrence would be critical to reduce the burden of disability in LGMDR. To date, there have been few large observational studies conducted that follow patients longitudinally to understand the natural history of LGMDR. However, the Global FKRP Registry has been established to support research in LGMDR9 [205]; as long-term data become available, this registry would offer important insights into the clinical trajectories for patients with this LGMD subtype.

The findings from this systematic review confirm that there is variability in age at LGMD onset across subtypes and provide novel details about LOA. Of note, the frequencies of individual subtypes within adult-, late childhood-, and early childhood-onset categories may be driven by data availability rather than true incidence, as some subtypes had considerably more patients with age at onset reported (e.g. LGMDR2/MM). Nevertheless, patients with LGMDR12 had onset predominantly in adulthood, which corresponds with previous reports that LGMD12 is characterized by late-onset lower limb weakness [206]. In comparison, patients with LGMDR3-6 frequently had early childhood-onset disease, similar to what was observed in a large cohort of patients with sarcoglycanopathy [8]. Variability in the occurrence of LOA was observed across LGMDR subtypes and across age at onset categories. LOA was more common among patients with early childhood- (71%) compared to late childhood- (59%) and adult-onset disease (52%). As ambulatory assessments were more frequent among patients with early childhood-onset disease, data on the occurrence and age at LOA may be more robust in this group than for those with adult-onset disease.

Regardless of age at disease onset, patients with LGMDR3-6 generally had the earliest LOA among the subtypes (at 32 years among adult-onset, 22 years among late childhood-onset, 17 years among early childhood-onset disease). Patients with early childhood-onset disease tended to have faster progression to LOA than those with late childhood- or adult-onset disease, particularly in LGMDR9. This observation is consistent with the findings for LGMDR3-5 from the European Sarcoglycanopathy Consortium, which reported that patients with disease onset before age 10 experienced significantly faster progression to ambulation loss than those with onset after 10 years of age [8].

Given the inclusion of case report data in this systematic review, limitations to study findings include that case report data are prone to bias as investigators may have different motivations for their analyses. In particular, there may be a bias of reporting more severe patients as case studies of the disease. For example, patients who had ambulatory function assessments likely had issues with ambulation in the first place that would prompt such an assessment. Moreover, given the cross-sectional nature of data included in this review, the reported absence of a clinical outcome such as LOA in a patient may not necessarily mean that this patient would not experience LOA subsequently. The small numbers of patients with LGMDR12 identified with LOA may be a consequence of such limitations, as these patients have a later onset of weakness and ambulation loss. In addition, as LGMDR12 was not characterized as a separate entity until recently [44], data availability for these patients may have been particularly affected. Large registries or observational studies that follow patients over time would be valuable to better characterize the natural history of LGMDR, as well as the prevalence of the different subtypes. Large observational studies would also be important for understanding other factors that may influence disease progression, such as socioeconomic characteristics and access to medical care, which are difficult to capture in a systematic review. For the present analyses, data availability was limited as patients were considered eligible only if both their age at LGMD onset and the age at which ambulation was assessed was reported. Therefore, the small numbers of patients meeting these criteria for some subtypes may limit the generalizability of these findings. However, given the rarity of some subtypes such as LGMDR6, small sample sizes were expected. Among included patients, it was often unclear whether they presented symptomatically, were asymptomatic but diagnosed as a result of early recognition through genetic screening, or were asymptomatic and diagnosed when they become symptomatic. As a result, the reported age at disease onset, and consequently the calculated time from onset to LOA, may reflect different experiences for different patients, even for the same LGMDR subtype. Selection and reporting bias may also have resulted in ambulatory status being preferentially investigated among those who were experiencing severe mobility difficulties or who eventually progressed to LOA, which would further impact generalizability. The frequency of LOA among patients with adult-onset disease may particularly be impacted by such biases given the challenge of capturing data for patients with mild disease. Despite the potential for overestimating the frequency of LOA for LGMDR from this review, the differences in frequency and age at LOA observed between subtypes may still hold true, e.g. that patients with sarcoglycanopathies experience faster progression to LOA than other subtypes. Although overall estimates of mean age at onset and age at LOA were determined for patients with adult-, late childhood-, and early childhood-onset LGMDR, data according to subtype may be more meaningful within these categories given the clinical heterogeneity across subtypes.

## CONCLUSIONS

Using currently available data, this review illustrated that LOA occurred most commonly among LGMDR patients with early childhood-onset disease, and least commonly among those with adult-onset disease. Moreover, patients with early childhood-onset disease tended to have faster progression to LOA than those with late childhood- or adult-onset disease, suggesting that age at disease onset may be an important risk factor for disease progression. While this systematic review was informed by patient-level data including case report data, which has its caveats such as reporting bias, it provides an in-depth assessment of patients with LGMDR across multiple countries and is a valuable addition to the literature for this rare disease. To complement and strengthen these findings, large registries such as the Global FKRP Registry or observational studies that follow patients over time would be important for better characterizing the natural history of LGMDR. Despite limited data, these findings provide a greater understanding of progression to LOA by subtype in LGMDR, which may help inform clinical trial design and provide a basis for natural history studies.

## Supplementary Material

Supplementary Material
