# Supplementary material for: Progression to Loss of Ambulation Among Patients with Autosomal Recessive Limb-girdle Muscular Dystrophy: A Systematic Review
Source: J Neuromuscul Dis. 2022 Jul 1;9(4):477–92. doi: 10.3233/JND-210771 (PMC9398075; doi:10.3233/JND-210771)
Supplement: Supplementary Material [file jnd-9-jnd210771-s001.docx]

SUPPLEMENTARY MATERIALS

**Supplementary Table 1. Scope of the literature review**

| **Population** | Individuals with LGMDR |
| --- | --- |
| **Exposures/Comparators** | Subgroups:   - Subtype/genotype   - Sarcoglycanopathies (LGMDR3-6)   - Dysferlinopathies (LGMDR2/MM)   - Calpainopathy (LGMDR1)   - FKRP-related dystroglycanopathy (LGMDR9)   - Anoctaminoapthy (LGMDR12) |
| **Outcomes** | Clinical measures:   - Serum creatinine kinase levels - MRI - ECG - Echocardiogram   Functional measures:   - Myometry measures - Muscle strength and functional testing - 6MWD - MMT - Brooke scale - AMS - Pulmonary function (e.g. FVC, FEV1) - ADL (e.g. Barthel Index) - Pain and fatigue (e.g. VAS) - General or LGMD-specific health-related quality of life (HRQoL) measures (e.g. Short Form-36, ACTIVLIM)   Longer-term outcomes, including but not limited to:   - LGMD disease progression by serial functional measures over time - Key clinical outcomes (e.g. need for wheelchair, need for ventilation, ambulation difficulties, heart failure, respiratory failure) - Mortality |
| **Study Design** | Epidemiologic (prospective or retrospective) studies  Randomized controlled trials  Case series (n>5)  Case reports |

**Abbreviations:** 6MWD, 6-minute walk distance; ADL, activities of daily living; AMS, average muscle score; ECG, electrocardiograph; FEV1, forced expiratory volume in 1 second; FKRP, fukutin-related protein; FVC, forced vital capacity; HRQoL, health-related quality of life; LGMDR, autosomal recessive limb girdle muscular dystrophy; MM, Miyoshi myopathy; MMT, manual muscle testing; MRI, magnetic resonance imaging; VAS, Visual Analog Scale.

**Supplementary Table 2. Characteristics of studies contributing patient data to the analysis**

| **Author, year** | **Study design** | **Country / Region** | **Subtypes** | **Number of patients** | **Study period / duration** |
| --- | --- | --- | --- | --- | --- |
| Aharoni et al, 2011 [1] | Case report | Israel | LGMDR2 | 1 | NR |
| Akarsu et al, 2018 [2] | Case report | Turkey | LGMDR12 | 1 | NR |
| Alavi et al, 2017 [3] | Retrospective | Iran | LGMDR3-6 | 25 | 2010-2015 |
| Algahtani et al, 2018 [4] | Case report | Saudi Arabia | LGMDR2 | 1 | NR |
| Alhamidi et al, 2017 [5] | Retrospective | Norway | LGMDR9 | 25 | NR |
| Al-Harbi et al, 2016 [6] | Case report | Saudi Arabia | LGMDR3 | 1 | NR |
| Angelini et al, 1998 [7] | Case report | Italy | LGMDR5 | 2 | NR |
| Angelini et al, 2010 [8] | Retrospective | Italy | LGMDR1, LGMDR2/MM | 43 | NR |
| Angelini et al, 2011 [9] | Case report | Italy | LGMDR2/MM | 2 | NR |
| Aoki et al, 2001 [10] | Retrospective | USA | MM | 24 | NR |
| Argov et al, 2000 [11] | Retrospective | Libya | LGMDR2 | 29 | 1997-98 |
| Arrigoni et al, 2018 [12] | Prospective | Italy | LGMDR1, LGMDR2 | 20 | NR |
| Bandyopadhyay et al, 2018 [13] | Case report | USA | LGMDR2 | 1 | NR |
| Barresi et al, 2000 [14] | Case report | Italy | LGMDR4 | 2 | NR |
| Baumeister et al, 2009 [15] | Case report | Germany | LGMDR5 | 1 | NR |
| Bhatt et al, 2019 [16] | Case report | India | LGMDR1 | 1 | NR |
| Bird et al, 2008 [17] | Case report | USA | LGMDR1 | 1 | NR |
| Blackburn et al, 2017 [18] | Case report | USA | LGMDR12 | 1 | NR |
| Boito et al, 2005 [19] | Retrospective | Italy | LGMDR9 | 20 | NR |
| Bonnemann et al, 1996 [20] | NR | Brazil | LGMDR4 | 7 | NR |
| Bouquet et al, 2012 [21] | Case report | France | LGMDR12 | 2 | NR |
| Burke et al, 2010 [22] | Case report | UK | LGMDR1 | 1 | NR |
| Cagliani et al, 2001 [23] | Case report | Italy | LGMDR4 | 1 | NR |
| Cagliani et al, 2003 [24] | NR | Italy | LGMDR2, MM | 7 | NR |
| Cagliani et al, 2005 [25] | Retrospective | Italy | LGMDR2/MM | 25 | NR |
| Cai et al, 2019 [26] | Retrospective | China | LGMDR12 | 5 | 2005-2018 |
| Calvo et al, 2000 [27] | NR | Spain | LGMDR5 | 10 | NR |
| Carman et al, 2018 [28] | Case report | Turkey | LGMDR2 | 1 | NR |
| Catteruccia et al, 2018 [29] | Case report | Italy | LGMDR3 | 2 | NR |
| Chourasia et al, 2017 [30] | Case report | USA | LGMDR9 | 1 | NR |
| Connolly et al, 1998 [31] | Case report | USA | LGMDR5 | 1 | NR |
| D'Amico et al, 2008 [32] | Case report | Italy | LGMDR9 | 1 | NR |
| Darin et al, 2007 [33] | Case report | Sweden | LGMDR9 | 2 | NR |
| de Albuquerque et al, 2015 [34] | Retrospective | Brazil | LGMDR1 | 6 | 3 years |
| de Paula et al, 2003 [35] | NR | Brazil | LGMDR9 | 16 | NR |
| Dicapua et al, 2014 [36] | Case report | USA | LGMDR5 | 2 | NR |
| Diers et al, 2007 [37] | Case report | Germany | LGMDR2 | 1 | NR |
| Dincer et al, 2000 [38] | Cross-sectional | Turkey | LGMDR1, LGMDR2, LGMDR3-6 | 25 | NR |
| Diniz et al, 2014 [39] | Case report | Turkey | LGMDR3 | 1 | NR |
| Diniz et al, 2015 [40] | Case report | Turkey | LGMDR4 | 1 | NR |
| Dirik et al, 2001 [41] | Case report | Turkey | LGMDR1 | 1 | NR |
| Driss et al, 2000 [42] | NR | Tunisia | LGMDR9 | 13 | NR |
| Fanin et al, 2003 [43] | Case series | Italy | LGMDR4 | 6 | NR |
| Fanin et al, 2004 [44] | Retrospective | Italy | LGMDR1 | 58 | NR |
| Fatehi et al, 2015 [45] | Prospective | Iran | LGMDR2 | 16 | NR |
| Fayssoil et al, 2018 [46] | Retrospective | France | LGMDR3-6 | 10 | NR |
| Feng et al, 2018 [47] | Cross-sectional | China | LGMDR1 | 32 | 2006-2016 |
| Figarella-Branger et al, 2002 [48] | Retrospective | France | LGMDR1, LGMDR3 | 5 | NR |
| Fischer et al, 2005 [49] | NR | Germany | LGMDR1, LGMDR2, LGMDR3, LGMDR9 | 17 | NR |
| Frosk et al, 2005 [50] | Case report | Canada | LGMDR9 | 3 | NR |
| Fu et al, 2016 [51] | Prospective | China | LGMDR9 | 12 | 2007-2016 |
| Fuschillo et al, 2010 [52] | Case report | Italy | LGMDR2/MM | 1 | NR |
| Gaul et al, 2006 [53] | Prospective | Germany | LGMDR9 | 9 | NR |
| Gemelli et al, 2017 [54] | Case report | Italy | LGMDR12 | 1 | NR |
| Ghafouri-Fard et al, 2017 [55] | Case report | Iran | LGMDR4 | 2 | NR |
| Ginjaar et al, 2000 [56] | Prospective | Netherlands | LGMDR3-5 | 8 | NR |
| Gomez-Andres et al, 2019 [57] | NR | Chile | LGMDR2/MM | 33 | 2011-2017 |
| Guglieri et al, 2008 [58] | Retrospective | Italy | LGMDR1, LGMDR2, LGMDR3-6, LGMDR9 | 181 | NR |
| Gulati et al, 2003 [59] | Case report | India | LGMDR5 | 1 | NR |
| Habig et al, 2017 [60] | Case report | Germany | LGMDR1 | 1 | NR |
| Hanisch et al, 2007 [61] | Retrospective | Germany | LGMDR1 | 9 | NR |
| Heikali et al, 2017 [62] | Case report | USA | LGMDR9 | 1 | NR |
| Hicks et al, 2011 [63] | Case series | UK, Germany | LGMDR12 | 20 | NR |
| Hollingsworth et al, 2013 [64] | Prospective | UK | LGMDR9 | 10 | NR |
| Hong et al, 2011 [65] | Case report | China | LGMDR9 | 2 | NR |
| Illa et al, 2007 [66] | Case report | Spain | LGMDR2 | 5 | NR |
| Izzedine et al, 2006 [67] | Case report | France | LGMDR2 | 1 | NR |
| Jenne et al, 2005 [68] | Case report | USA | LGMDR1 | 1 | 1995-2004 |
| Jethwa et al, 2013 [69] | Case report | UK | LGMDR2 | 1 | NR |
| Jomaa et al, 2009 [70] | Case report | Tunisia | LGMDR5 | 1 | NR |
| Kang et al, 2007 [71] | Prospective | USA | LGMDR9 | 7 | NR |
| Kapoor et al, 2005 [72] | Case report | India | LGMDR4 | 1 | NR |
| Kefi et al, 2008 [73] | Case report | Tunisia | LGMDR9 | 4 | NR |
| Khadilkar et al, 2008 [74] | Case report | India | LGMDR2/MM | 2 | 22 years |
| Khaiboullina et al, 2017 [75] | Case report | Russia | LGMDR2 | 3 | NR |
| Kida et al, 2015 [76] | Case report | Japan | LGMDR12 | 1 | 2004 |
| Klinge et al, 2008 [77] | Case report | UK | LGMDR2/MM | 1 | NR |
| Kocak Eker et al, 2019 [78] | Case report | Turkey | LGMDR6 | 1 | NR |
| Koken et al, 2018 [79] | Case report | Turkey | LGMDR9 | 1 | NR |
| Krishnaiah et al, 2016 [80] | Case report | USA | LGMDR3 | 1 | NR |
| Lanzillo et al, 2006 [81] | Case report | Italy | LGMDR1 | 1 | NR |
| Lerario et al, 2010 [82] | Case report | Italy | LGMDR2/MM | 2 | NR |
| Li et al, 2014 [83] | Case report | China | LGMDR2 | 1 | NR |
| Liewluck et al, 2012 [84] | Case report | USA | LGMDR1 | 1 | NR |
| Liewluck et al, 2013 [85] | Retrospective | USA | LGMDR12 | 7 | 1998-2012 |
| Lin et al, 2007 [86] | Case report | Tiawan | LGMDR9 | 1 | NR |
| Lindberg et al, 2011 [87] | Case report | Sweden | LGMDR9 | 5 | 2 years |
| Little et al, 2013 [88] | Case report | USA | LGMDR12 | 1 | NR |
| Lokken et al, 2016 [89] | Cross-sectional | Denmark | LGMDR9 | 11 | NR |
| Lomma et al, 2018 [90] | Case report | Australia | LGMDR1 | 1 | NR |
| Lu et al, 2019 [91] | Case report | China | LGMDR3 | 1 | NR |
| Margeta et al, 2009 [92] | Case report | USA | LGMDR9 | 2 | NR |
| Martinez-Thompson et al, 2018 [93] | Case report | USA | LGMDR1 | 1 | NR |
| Mashiah et al, 2016 [94] | Case report | Israel | LGMDR2 | 1 | NR |
| Matsubara et al, 2007 [95] | Case report | Japan | LGMDR1 | 2 | NR |
| McMillan et al, 2009 [96] | Case report | Canada | LGMDR5 | 1 | NR |
| McNally et al, 1996 [97] | Case report | Brazil | LGMDR5 | 11 | NR |
| Mendell et al, 2010 [98] | RCT | USA | LGMDR3 | 6 | 2 years |
| Mercuri et al, 2003 [99] | Prospective | UK | LGMDR9 | 22 | NR |
| Meznaric-Petrusa et al, 2009 [100] | Case report | Slovenia | LGMDR3 | 1 | NR |
| Miskew Nichols et al, 2018 [101] | Case report | Canada | LGMDR9 | 1 | NR |
| Mojbafan et al, 2016 [102] | Case report | Iran | LGMDR3 | 5 | NR |
| Moody et al, 2013 [103] | Case report | USA | LGMDR2 | 1 | NR |
| Muller et al, 2005 [104] | Case report | Germany | LGMDR9 | 3 | NR |
| Mustafa et al, 2014 [105] | Case report | Bangladesh | LGMDR4 | 1 | NR |
| Nagashima et al, 2004 [106] | Case report | Japan | LGMDR2, MM | 1 | NR |
| Negrao et al, 2012 [107] | Case report | Portugal | LGMDR12 | 3 | NR |
| Nemes et al, 2017 [108] | Case report | Hungary | LGMDR1 | 1 | NR |
| Neto et al, 2018 [109] | Case report | NR | LGMDR1 | 2 | NR |
| Nowak et al, 2000 [110] | Case report | Australia | LGMDR5 | 2 | NR |
| Oexle et al, 1996 [111] | Case report | Bulgaria | LGMDR5 | 2 | NR |
| Oflazer et al, 2009 [112] | Case report | Turkey | LGMDR1 | 1 | NR |
| Okahashi et al, 2008 [113] | Case report | Japan | LGMDR2 | 1 | NR |
| Okere et al, 2013 [114] | Case report | USA | LGMDR1 | 1 | NR |
| Oliveira Santos et al, 2018 [115] | Case report | Guinea-Bissau | LGMDR1 | 2 | NR |
| Paradas et al, 2010 [116] | Retrospective | Spain | LGMD2, MM | 26 | NR |
| Park et al, 2012 [117] | Retrospective | Korea | LGMDR2, MM | 28 | NR |
| Passos-Bueno et al, 1996 [118] | Retrospective | Brazil | LGMDR3 | 63 | NR |
| Patel et al, 2017 [119] | Case report | USA | LGMDR2/MM | 2 | NR |
| Peddareddygari et al, 2018 [120] | Case report | USA | LGMDR12 | 1 | NR |
| Pena et al, 2010 [121] | Case report | USA | LGMDR5 | 1 | NR |
| Penisson-Besnier et al, 1998 [122] | Case report | France | LGMDR1 | 2 | NR |
| Penttila et al, 2012 [123] | Retrospective | Finland | LGMDR12 | 25 | NR |
| Peric et al, 2019 [124] | NR | Serbia | LGMDR1 | 19 | 2008-2017 |
| Petersen et al, 2015 [125] | Retrospective | Switzerland | LGMDR2 | 13 | 1989-2015 |
| Pimentel et al, 2008 [126] | Case report | Brazil | LGMDR2 | 3 | NR |
| Pizzanelli et al, 2006 [127] | Case report | Italy | LGMDR1 | 1 | NR |
| Pollitt et al, 2001 [128] | Retrospective | UK | LGMDR1 | 13 | NR |
| Poppe et al, 2003 [129] | Retrospective | UK | LGMDR9 | 16 | NR |
| Prahm et al, 2017 [130] | Case report | Denmark | LGMDR1 | 1 | NR |
| Prelle et al, 1998 [131] | NR | Italy | LGMDR3-6 | 12 | NR |
| Prelle et al, 2003 [132] | Case series | Italy | LGMDR2/MM | 6 | NR |
| Quick et al, 2015 [133] | NR | Germany | LGMDR1 | 10 | January-September 2013 |
| Quinlivan et al, 1997 [134] | Case report | UK | LGMDR3 | 1 | NR |
| Rasmussen et al, 2014 [135] | Retrospective | Norway | LGMDR9 | 17 | 2004-2012 |
| Rekik et al, 2019 [136] | Case report | Tunisia | LGMDR1 | 1 | NR |
| Renard et al, 2012 [137] | Case report | France | LGMDR9 | 1 | NR |
| Ro et al, 2004 [138] | Prospective | Taiwan | MM | 4 | NR |
| Rosales et al, 2010 [139] | Retrospective | USA | LGMDR2 | 21 | NR |
| Rosales et al, 2011 [140] | Prospective | USA | LGMDR2, LGMDR9 | 16 | October 2006-January 2009 |
| Saenz et al, 2011 [141] | Case report | Spain | LGMDR1 | 2 | NR |
| Sahai et al, 2019 [142] | Case report | USA | LGMDR1 | 1 | NR |
| Sarkozy et al, 2012 [143] | NR | UK | LGMDR12 | 25 | NR |
| Sarkozy et al, 2013 [144] | Retrospective | UK | LGMDR12 | 44 | NR |
| Schessl et al, 2012 [145] | Case report | Germany | LGMDR12 | 4 | NR |
| Schneider et al, 2014 [146] | Case report | Germany | LGMDR12 | 1 | NR |
| Schutz et al, 2017 [147] | Case report | UK | LGMDR1 | 1 | NR |
| Sczesny-Kaiser et al, 2017 [148] | Case report | Germany | LGMDR1, LGMDR9 | 2 | NR |
| Selva-O'Callaghan et al, 2006 [149] | Case report | Spain | LGMDR2 | 1 | NR |
| Seong et al, 2016 [150] | Prospective | Korea | LGMDR1, LGMDR2 | 13 | NR |
| Sharma et al, 2004 [151] | Retrospective and prospective | India | LGMDR3-6 | 13 | 1998-2004 |
| Shouman et al, 2018 [152] | Case report | USA | LGMDR12 | 2 | NR |
| Strafella et al, 2019 [153] | Case report | Italy | LGMDR1 | 1 | NR |
| Svahn et al, 2015 [154] | Case report | France | LGMDR9 | 1 | NR |
| Szymanska et al, 2014 [155] | Case report | Poland | LGMDR2 | 1 | NR |
| Takahashi et al, 2003 [156] | Retrospective | Japan | MM | 25 | NR |
| Takahashi et al, 2006 [157] | Case report | Japan | LGMDR2 | 1 | NR |
| Takahashi et al, 2019 [158] | Case report | Japan | LGMDR2 | 1 | NR |
| Takano et al, 2000 [159] | Case report | Japan | LGMDR5 | 2 | 1991-1998 |
| Tarnopolsky et al, 2015 [160] | Case report | USA | LGMDR3 | 2 | NR |
| Tasca et al, 2014 [161] | Case report | Italy | LGMDR12 | 1 | NR |
| Tasca et al, 2018 [162] | Prospective | Europe | LGMDR3-6 | 69 | NR |
| Tetreault et al, 2011 [163] | Retrospective | Canada | LGMDR3 | 9 | NR |
| Todorova et al, 2007 [164] | Retrospective | Bulgaria | LGMDR1 | 22 | NR |
| Topaloglu et al, 1997 [165] | Prospective | Turkey | LGMDR1 | 10 | NR |
| Tsuburaya et al, 2011 [166] | Case report | Japan | LGMDR1 | 1 | NR |
| Ueyama et al, 2001 [167] | NR | Japan | LGMDR2, MM | 5 | NR |
| Umakhanova et al, 2017 [168] | Prospective | Russia | LGMDR2 | 12 | NR |
| Vainzof et al, 2000 [169] | NR | Brazil | LGMDR3 | 3 | NR |
| Van der Kooi et al, 1998 [170] | Case report | The Netherlands | LGMDR5 | 5 | NR |
| Van der Kooi et al, 2013 [171] | Retrospective | The Netherlands | LGMDR12 | 13 | >15 years |
| Viloria-Alebesque et al, 2019 [172] | Case report | Spain | LGMDR1 | 1 | NR |
| Vissing et al, 2014 [173] | Prospective | Denmark | LGMDR12 | 6 | NR |
| Von Der Hagen et al, 2006 [174] | Case report | Germany | LGMDR9 | 2 | NR |
| Von Guionneau et al, 2018 [175] | Case report | UK | LGMDR9 | 1 | NR |
| Vorgerd et al, 2001 [176] | Case report | Germany | LGMDR5 | 1 | NR |
| Wahbi et al, 2008 [177] | Prospective | France | LGMDR9 | 23 | NR |
| Walsh et al, 2011 [178] | Case report | Ireland | LGMDR2 | 1 | NR |
| Walter et al, 2003 [179] | Case report | Germany | LGMDR2/MM | 4 | NR |
| Walter et al, 2004 [180] | Case report | Germany | LGMDR3 | 2 | NR |
| Walter et al, 2013 [181] | RCT | Germany | LGMDR2, MM | 23 | 2003-2008 |
| Wang et al, 2015 [182] | Case report | Taiwan | LGMDR1 | 1 | NR |
| Wang et al, 2018 [183] | Case report | China | LGMDR9 | 2 | NR |
| Weiler et al, 1996 [184] | Retrospective | Canada | LGMDR2, MM | 9 | NR |
| Willis et al, 2014 [185] | Cross-sectional | Europe | LGMDR9 | 38 | NR |
| Wong-Kisiel et al, 2010 [186] | Case report | USA | LGMDR4 | 2 | NR |
| Woudt et al, 2016 [187] | Prospective | Chile | LGMDR2, MM | 31 | 2009-2012 |
| Xi et al, 2014 [188] | Prospective | China | LGMDR2/MM | 29 | 2005-2009 |
| Xie et al, 2018 [189] | Prospective | China | LGMDR9 | 10 | NR |
| Xie et al, 2019 [190] | Prospective | China | LGMDR3-6 | 25 | January 2013-August 2018 |
| Xu et al, 2018 [191] | Case report | China | LGMDR2 | 1 | NR |
| Yilmaz et al, 2009 [192] | Case report | Germany | LGMDR5 | 1 | NR |
| Ylikallio et al, 2016 [193] | Retrospective | Finland | LGMDR12 | 14 | NR |
| Yu et al, 2017 [194] | Prospective | China | LGMDR1, LGMDR2, LGMDR3, LGMDR9, LGMDR12 | 92 | January 2013-May 2015 |
| Yuksel et al, 2018 [195] | Case report | NR | LGMDR4 | 1 | NR |

**Abbreviations:** LGMD, limb girdle muscular dystrophy; MM, Miyoshi myopathy; NR, not reported; RCT, randomized controlled trial, UK, United Kingdom; USA, United States of America.

SUPPLEMENTARY REFERENCES

[1] Aharoni S, Sadeh M, Silver EL, Straussberg R. Dysferlinopathy and very-long-chain acyl coenzyme a dehydrogenase deficiency segregating in the same family. Israel Medical Association Journal. 2011;13(10):632-4.

[2] Akarsu EO, Kotan D, Alemdar M, Tunc A. Myopathy and epilepsy: A rare phenotype in limb-girdle muscular dystrophy 2l associated with ANO5 mutation. Acta Myologica. 2018;37 (2):161.

[3] Alavi A, Esmaeili S, Nilipour Y, Nafissi S, Tonekaboni SH, Zamani G, et al. LGMD2E is the most common type of sarcoglycanopathies in the Iranian population. Journal of Neurogenetics. 2017;31(3):161-9.

[4] Algahtani H, Shirah B, Alassiri AH, Habib BA, Almuhanna R, Ahamed MF. Limb-girdle muscular dystrophy type 2B: An unusual cause of proximal muscular weakness in Saudi Arabia. Journal of Back and Musculoskeletal Rehabilitation. 2018;31(5):999-1004.

[5] Alhamidi M, Brox V, Stensland E, Liset M, Lindal S, Nilssen O. Limb girdle muscular dystrophy type 2I: No correlation between clinical severity, histopathology and glycosylated alpha-dystroglycan levels in patients homozygous for common FKRP mutation. Neuromuscular Disorders. 2017;27(7):619-26.

[6] Al-Harbi KM, Abdallah AM. LGMD2D syndrome: The importance of clinical and molecular genetics in patient and family management. Neuroendocrinology Letters. 2016;37(4):277-81.

[7] Angelini C, Fanin M, Menegazzo E, Freda MP, Duggan DJ, Hoffman EP. Homozygous alpha-sarcoglycan mutation in two siblings: One asymptomatic and one steroid-responsive mild limb-girdle muscular dystrophy patient. Muscle and Nerve. 1998;21(6):769-75.

[8] Angelini C, Nardetto L, Borsato C, Padoan R, Fanin M, Nascimbeni AC, et al. The clinical course of calpainopathy (LGMD2A) and dysferlinopathy (LGMD2B). Neurological Research. 2010;32(1):41-6.

[9] Angelini C, Peterle E, Gaiani A, Bortolussi L, Borsato C. Dysferlinopathy course and sportive activity: Clues for possible treatment. Acta Myologica. 2011;30(OCTOBER):127-32.

[10] Aoki M, Liu J, Richard I, Bashir R, Britton S, Keers SM, et al. Genomic organization of the dysferlin gene and novel mutations in Miyoshi myopathy. Neurology. 2001;57(2):271-8.

[11] Argov Z, Sadeh M, Mazor K, Soffer D, Kahana E, Eisenberg I, et al. Muscular dystrophy due to dysferlin deficiency in Libyan Jews. Clinical and genetic features. Brain. 2000;123(6):1229-37.

[12] Arrigoni F, De Luca A, Velardo D, Magri F, Gandossini S, Russo A, et al. Multiparametric quantitative MRI assessment of thigh muscles in limb-girdle muscular dystrophy 2A and 2B. Muscle and Nerve. 2018;58(4):550-8.

[13] Bandyopadhyay S. A novel phenotype of miyoshi myopathy or LGMD2B muscular dystrophy with early severe contractures and normal CK, simulating atypical bethlem myopathy. Muscle and Nerve. 2018;58 (Supplement 2):S67.

[14] Barresi R, Morandi L, Mora M, Di Blasi C, Negri T, Brugnoni R, et al. Disruption of heart sarcoglycan complex and severe cardiomyopathy caused by beta sarcoglycan mutations. Journal of Medical Genetics. 2000;37(2):102-7.

[15] Baumeister SK, Todorovic S, Milic-Rasic V, Dekomien G, Lochmuller H, Walter MC. Eosinophilic myositis as presenting symptom in gamma-sarcoglycanopathy. Neuromuscular Disorders. 2009;19(2):167-71.

[16] Bhatt AD, Shah K, Puvar A, Joshi CG, Joshi M. A case of limb girdle muscular dystrophy type 2A from India: Copy number variation analysis using targeted amplicon sequencing. Journal of Clinical and Diagnostic Research. 2019;13(4):GD01-GD2.

[17] Bird TD, Lipe HP, Steinbart EJ. Geriatric neurogenetics: Oxymoron or reality? Archives of Neurology. 2008;65(4):537-9.

[18] Blackburn PR, Selcen D, Jackson JL, Guthrie KJ, Cousin MA, Boczek NJ, et al. Early-onset limb-girdle muscular dystrophy-2L in a female athlete. Muscle and Nerve. 2017;55(5):E19-E21.

[19] Boito CA, Melacini P, Vianello A, Prandini P, Gavassini BF, Bagattin A, et al. Clinical and molecular characterization of patients with limb-girdle muscular dystrophy type 2I. Archives of Neurology. 2005;62(12):1894-9.

[20] Bonnemann CG, Passos-Bueno MR, McNally EM, Vainzof M, Moreira EDS, Marie SK, et al. Genomic screening for beta-sarcoglycan gene mutations: Missense mutations may cause severe limb-girdle muscular dystrophy type 2E (LGMD 2E). Human Molecular Genetics. 1996;5(12):1953-61.

[21] Bouquet F, Cossee M, Behin A, Deburgrave N, Romero N, Leturcq F, et al. Miyoshi-like distal myopathy with mutations in anoctamin 5 gene. Revue Neurologique. 2012.

[22] Burke G, Hillier C, Cole J, Sampson M, Bridges L, Bushby K, et al. Calpainopathy presenting as foot drop in a 41 year old. Neuromuscular Disorders. 2010;20(6):407-10.

[23] Cagliani R, Comi GP, Tancredi L, Sironi M, Fortunate F, Giorda R, et al. Primary beta-sarcoglycanopathy manifesting as recurrent exercise-induced myoglobinuria. Neuromuscular Disorders. 2001;11(4):389-94.

[24] Cagliani R, Fortunato F, Giorda R, Rodolico C, Bonaglia MC, Sironi M, et al. Molecular analysis of LGMD-2B and MM patients: Identification of novel DYSF mutations and possible founder effect in the Italian population. Neuromuscular Disorders. 2003;13(10):788-95.

[25] Cagliani R, Magri F, Toscano A, Merlini L, Fortunato F, Lamperti C, et al. Mutation finding in patients with dysferlin deficiency and role of the dysferlin interacting proteins annexin A1 and A2 in muscular dystrophies. Human mutation. 2005;26(3):283.

[26] Cai S, Gao M, Xi J, Liu Z, Yue D, Wu H, et al. Clinical spectrum and gene mutations in a Chinese cohort with anoctaminopathy. Neuromuscular Disorders. 2019.

[27] Calvo F, Teijeira S, Fernandez JM, Teijeiro A, Fernandez-Hojas R, Fernandez-Lopez XA, et al. Evaluation of heart involvement in gamma-sarcoglycanopathy (LGMD2C). A study of ten patients. Neuromuscular Disorders. 2000;10(8):560-6.

[28] Carman KB, Yimenicioglu S, Yarar C. Limb-girdle muscular distrophy type 2B: A case presented with abdominal and ankle pain. Acta Myologica. 2018;37 (2):160.

[29] Catteruccia M, Primiano G, Fattori F, Bertini E, Servidei S, D'Amico A. Exercise intolerance and myoglobinuria as presenting symptom of alpha-sarcoglycanopathy. Acta Myologica. 2018;37 (1):58.

[30] Chourasia N, Biliciler S. EDB hypertrophy associated with FKRP mutation in LGMD 21. Journal of Clinical Neuromuscular Disease. 2017;18 (Supplement 1):S11.

[31] Connolly AM, Pestronk A, Mehta S, Al-Lozi M. Primary alpha-sarcoglycan deficiency responsive to immunosuppression over three years. Muscle and Nerve. 1998;21(11):1549-53.

[32] D'Amico A, Petrini S, Parisi F, Tessa A, Francalanci P, Grutter G, et al. Heart transplantation in a child with LGMD2I presenting as isolated dilated cardiomyopathy. Neuromuscular Disorders. 2008;18(2):153-5.

[33] Darin N, Kroksmark AK, Ahlander AC, Moslemi AR, Oldfors A, Tulinius M. Inflammation and response to steroid treatment in limb-girdle muscular dystrophy 2I. European Journal of Paediatric Neurology. 2007;11(6):353-7.

[34] de Albuquerque MAV, Abath Neto O, da Silva FMA, Zanoteli E, Reed UC. Limb-girdle muscular dystrophy type 2A in Brazilian children. Arquivos de Neuro-Psiquiatria. 2015;73(12):993-7.

[35] de Paula F, Vieira N, Starling A, Yamamoto LU, Lima B, de Cassia Pavanello R, et al. Asymptomatic carriers for homozygous novel mutations in the FKRP gene: The other end of the spectrum. European Journal of Human Genetics. 2003;11(12):923-30.

[36] Dicapua D, Patwa H. Puerto rican founder mutation G787A in the SGCG Gene: A case report of 2 siblings with LGMD 2C. Journal of Clinical Neuromuscular Disease. 2014;15(3):105-7.

[37] Diers A, Carl M, Stoltenburg-Didinger G, Vorgerd M, Spuler S. Painful enlargement of the calf muscles in limb girdle muscular dystrophy type 2B (LGMD2B) with a novel compound heterozygous mutation in DYSF. Neuromuscular Disorders. 2007;17(2):157-62.

[38] Dincer PU, J. A.; Beckmann, J. S.; Topaloglu, H.; Akcoren, Z.; Demir, E.; Richard, I.; Sancak, O.; Kale, G.; Ozme, S.; Karaduman, A.; Tan, E. A cross section of autosomal recessive limb-girdle muscular dystrophies in 38 families. Journal of Medical Genetics. 2000;37(5):361-7.

[39] Diniz G, Tekgul H, Hazan F, Yararbas K, Tukun A. Sarcolemmal deficiency of sarcoglycan complex in an eighteen months old Turkish boy with a huge deletion in the beta sarcoglycan gene. Neuromuscular Disorders. 2014;24 (9-10):884-5.

[40] Diniz G, Tekgul H, Hazan F, Yararbas K, Tukun A. Sarcolemmal deficiency of sarcoglycan complex in an 18-month-old Turkish boy with a large deletion in the beta sarcoglycan gene. Balkan Journal of Medical Genetics. 2015;18(2):71-6.

[41] Dirik E, Aydin A, Kurul S, Sahin B. Limb girdle muscular dystrophy type 2A presenting with cardiac arrest. Pediatric Neurology. 2001;24(3):235-7.

[42] Driss A, Amouri R, Ben Hamida C, Souilem S, Gouider-Khouja N, Ben Hamida M, et al. A new locus for autosomal recessive limb-girdle muscular dystrophy in a large consanguineous Tunisian family maps to chromosome 19q13.3. Neuromuscular Disorders. 2000;10(4-5):240-6.

[43] Fanin M, Melacini P, Boito C, Pegoraro E, Angelini C. LGMD2E patients risk developing dilated cardiomyopathy. Neuromuscular Disorders. 2003;13(4):303-9.

[44] Fanin M, Fulizio L, Nascimbeni AC, Spinazzi M, Piluso G, Ventriglia VM, et al. Molecular diagnosis in LGMD2A: Mutation analysis of protein testing? Human Mutation. 2004;24(1):52-62.

[45] Fatehi F, Nafissi S, Urtizberea JA, Blanck-Labelle V, Levy N, Krahn M, et al. Dysferlinopathy in Iran: Clinical and genetic report. Journal of the Neurological Sciences. 2015;359(1-2):256-9.

[46] Fayssoil A, Nguyen LS, Ogna A, Meng P, Nardi O, Laforet P, et al. Effects of Home Mechanical Ventilation on Left Ventricular Function in Sarcoglycanopathies (Limb Girdle Muscular Dystrophies). American Journal of Cardiology. 2018;122(2):353-5.

[47] Feng X, Luo S, Li J, Yue D, Xi J, Zhu W, et al. Fatty infiltration evaluation and selective pattern characterization of lower limbs in limb-girdle muscular dystrophy type 2A by muscle magnetic resonance imaging. Muscle and Nerve. 2018;58(4):536-41.

[48] Figarella-Branger D, El-Dassouki M, Saenz A, Cobo AM, Malzac P, Tong S, et al. Myopathy with lobulated muscle fibers: Evidence for heterogeneous etiology and clinical presentation. Neuromuscular Disorders. 2002;12(1):4-12.

[49] Fischer D, Walter MC, Kesper K, Petersen JA, Aurino S, Nigro V, et al. Diagnostic value of muscle MRI in differentiating LGMD2I from other LGMDs. Journal of Neurology. 2005;252(5):538-47.

[50] Frosk P, Del Bigio MR, Wrogemann K, Greenberg CR. Hutterite brothers both affected with two forms of limb girdle muscular dystrophy: LGMD2H and LGMD2I. European Journal of Human Genetics. 2005;13(8):978-82.

[51] Fu X, Yang H, Wei C, Jiao H, Wang S, Yang Y, et al. FKRP mutations, including a founder mutation, cause phenotype variability in Chinese patients with dystroglycanopathies. Journal of Human Genetics. 2016;61(12):1013-20.

[52] Fuschillo S, Torrente Y, Balzano G. Severe respiratory and skeletal muscles involvement in a carrier of dysferlinopathy with chronic obstructive pulmonary disease. Respiratory Care. 2010;55(8):1091-3.

[53] Gaul C, Deschauer M, Tempelmann C, Vielhaber S, Klein HU, Heinze HJ, et al. Cardiac involvement in limb-girdle muscular dystrophy 2I: Conventional cardiac diagnostic and cardiovascular magnetic resonance. Journal of Neurology. 2006;253(10):1317-22.

[54] Gemelli C, Fiorillo C, Fabbri S, Cabona C, Zara F, Madia F, et al. A case of limb-girdle muscular dystrophy type 2L mimicking Dermatomyositis. Acta Myologica. 2017;36 (2):94.

[55] Ghafouri-Fard S, Hashemi Gorji F, Fardaei M, Miryounesi M. Limb girdle muscular dystrophy type 2E due to a novel large deletion in SGCB gene. Iranian Journal of Child Neurology. 2017;11(3):57-60.

[56] Ginjaar HB, Van Der Kooi AJ, Ceelie H, Kneppers ALJ, Van Meegen M, Barth PG, et al. Sarcoglycanopathies in Dutch patients with autosomal recessive limb girdle muscular dystrophy. Journal of Neurology. 2000;247(7):524-9.

[57] Gomez-Andres D, Diaz J, Munell F, Sanchez-Montanez A, Pulido-Valdeolivas I, Suazo L, et al. Disease duration and disability in dysfeRlinopathy can be described by muscle imaging using heatmaps and random forests. Muscle Nerve. 2019;59(4):436-44.

[58] Guglieri M, Magri F, D'Angelo MG, Prelle A, Morandi L, Rodolico C, et al. Clinical, molecular, and protein correlations in a large sample of genetically diagnosed Italian limb girdle muscular dystrophy patients. Human Mutation. 2008;29(2):258-66.

[59] Gulati S, Leekha S, Sharma MC, Kalra V. Gamma-Sarcoglycanopathy. Indian Pediatrics. 2003;40(11):1077-81.

[60] Habig K, Wurzer B, Gunther B, Schanzer A, Nolte D, Kramer HH. A slight change with a major impact-a report on a patient with limb girdle muscular dystrophy. Clinical Neurophysiology. 2017;128 (10):e354.

[61] Hanisch F, Muller CR, Grimm D, Xue L, Traufeller K, Merkenschlager A, et al. Frequency of calpain-3 c.550delA mutation in limb girdle muscular dystrophy type 2 and isolated hyperCKemia in German patients. Clinical Neuropathology. 2007;26(4):157-63.

[62] Heikali D, Bokhoor P. New onset cardiomyopathy and cardiogenic shock leading to a diagnosis of limbgirdle muscular dystrophy 2I. Journal of the American College of Cardiology. 2017;69 (11 Supplement 1):2352.

[63] Hicks D, Sarkozy A, Muelas N, Koehler K, Huebner A, Hudson G, et al. A founder mutation in Anoctamin 5 is a major cause of limb-girdle muscular dystrophy. Brain. 2011;134(1):171-82.

[64] Hollingsworth KG, Willis TA, Bates MGD, Dixon BJ, Lochmuller H, Bushby K, et al. Subepicardial dysfunction leads to global left ventricular systolic impairment in patients with limb girdle muscular dystrophy 2I. European Journal of Heart Failure. 2013;15(9):986-94.

[65] Hong D, Zhang W, Wang W, Wang Z, Yuan Y. Asian patients with limb girdle muscular dystrophy 2I (LGMD2I). Journal of Clinical Neuroscience. 2011;18(4):494-9.

[66] Illa I, De Luna N, Dominguez-Perles R, Rojas-Garcia R, Paradas C, Palmer J, et al. Symptomatic dysferlin gene mutation carriers: Characterization of two cases. Neurology. 2007;68(16):1284-9.

[67] Izzedine H, Brocheriou I, Eymard B, Le Charpentier M, Romero NB, LeNaour G, et al. Loss of Podocyte Dysferlin Expression Is Associated With Minimal Change Nephropathy. American Journal of Kidney Diseases. 2006;48(1):143-50.

[68] Jenne DE, Kley RA, Vorgerd M, Schroder JM, Weis J, Reimann H, et al. Limb girdle muscular dystrophy in a sibling pair with a homozygous Ser606Leu mutation in the alternatively spliced IS2 region of calpain 3. Biological Chemistry. 2005;386(1):61-7.

[69] Jethwa H, Jacques TS, Gunny R, Wedderburn LR, Pilkington C, Manzur AY. Limb girdle muscular dystrophy type 2B masquerading as inflammatory myopathy: Case report. Pediatric Rheumatology. 2013;11 (1) (no pagination)(19).

[70] Jomaa R, Star MH, Mhenni SY, Jenzri S, Jerbi S, Zantour B, et al. Isolated neurosarcoidosis revealed by diabetes insipidus, visual loss and diplopia in a child patient: A diagnostic problem. Clinical Pediatric Endocrinology. 2009;18(1):51-4.

[71] Kang PB, Feener CA, Estrella E, Thorne M, White AJ, Darras BT, et al. LGMD2I in a North American population. BMC Musculoskeletal Disorders. 2007;8 (no pagination)(115).

[72] Kapoor S, Tatke M, Aggarwal S, Gupta A. Beta-sarcoglycanopathy. Indian Journal of Pediatrics. 2005;72(1):71-4.

[73] Kefi M, Amouri R, Chabrak S, Mechmeche R, Hentati F. Variable cardiac in involvement in Tunisian siblings harboring FKRP gene mutations. Neuropediatrics. 2008;39(2):113-5.

[74] Khadilkar SV, Singh RK, Agarwal P, Krahn M, Levy N. Twenty-two year follow-up of an Indian family with dysferlinopathy-clinical, immunocytochemical, western blotting and genetic features. Neurology India. 2008;56(3):388-90.

[75] Khaiboullina SF, Martynova EV, Bardakov SN, Mavlikeev MO, Yakovlev IA, Isaev AA, et al. Serum Cytokine Profile in a Patient Diagnosed with Dysferlinopathy. Case Reports in Medicine. 2017;2017 (no pagination)(3615354).

[76] Kida H, Sano K, Yorita A, Miura S, Ayabe M, Hayashi Y, et al. First Japanese case of muscular dystrophy caused by a mutation in the anoctamin 5 gene. Neurology and Clinical Neuroscience. 2015;3(4):150-2.

[77] Klinge L, Dean AF, Kress W, Dixon P, Charlton R, Muller JS, et al. Late onset in dysferlinopathy widens the clinical spectrum. Neuromuscular Disorders. 2008;18(4):288-90.

[78] Kocak Eker H, Balasar O. Limb-girdle muscular dystrophy: A family with three cases. Gazi Medical Journal. 2019;30 (1):P93.

[79] Koken OY, Kayilioglu H, Sel CG, Oztoprak U, Talim B, Yuksel D. A case of limb-girdle muscular dystrophy 2I due to FKRP mutation presenting as acute myositis. Acta Myologica. 2018;37 (2):161-2.

[80] Krishnaiah B, Lee JJ, Wicklund MP, Kaur D. Young girl presenting with exercise-induced myoglobinuria. Muscle and Nerve. 2016;54(1):161-4.

[81] Lanzillo R, Aurino S, Fanin M, Aguennoz M, Vitale F, Fiorillo C, et al. Early onset calpainopathy with normal non-functional calpain 3 level. Developmental Medicine and Child Neurology. 2006;48(4):304-6.

[82] Lerario A, Cogiamanian F, Marchesi C, Belicchi M, Bresolin N, Porretti L, et al. Effects of rituximab in two patients with dysferlin-deficient muscular dystrophy. BMC Musculoskeletal Disorders. 2010;11:157.

[83] Li F, Yin G, Xie Q, Shi G. Late-onset dysferlinopathy presented as "liver Enzyme" abnormalities: A technical note. Journal of Clinical Rheumatology. 2014;20(5):275-7.

[84] Liewluck T, Goodman BP. Late-onset axial myopathy and camptocormia in a calpainopathy carrier. Journal of Clinical Neuromuscular Disease. 2012;13(4):209-13.

[85] Liewluck T, Winder TL, Dimberg EL, Crum BA, Heppelmann CJ, Wang Y, et al. ANO5-muscular dystrophy: Clinical, pathological and molecular findings. European Journal of Neurology. 2013;20(10):1383-9.

[86] Lin YC, Murakami T, Hayashi YK, Nishino I, Nonaka I, Yuo CY, et al. A novel FKRP gene mutation in a Taiwanese patient with limb-girdle muscular dystrophy 2I. Brain and Development. 2007;29(4):234-8.

[87] Lindberg C, Sixt C, Oldfors A. Episodes of exercise-induced dark urine and myalgia in LGMD 2I. Acta Neurologica Scandinavica. 2011.

[88] Little AA, McKeever PE, Gruis KL. Novel mutations in the anoctamin 5 gene (ANO5) associated with limb-girdle muscular dystrophy 2L. Muscle and Nerve. 2013;47(2):287-91.

[89] Lokken N, Hedermann G, Thomsen C, Vissing J. Contractile properties are disrupted in Becker muscular dystrophy, but not in limb girdle type 2I. Annals of Neurology. 2016;80(3):466-71.

[90] Lomma C, Ransom D. Chemotherapy dosing and toxicity in a patient with muscular dystrophy. Cancer Reports. 2018;1 (2) (no pagination)(e1106).

[91] Lu Y, Song X, Ji G, Wu H, Li D, Sun S. Identification of a novel SGCA missense mutation in a case of limb-girdle muscular dystrophy 2D with the absence of four sarcoglycan proteins. Neuropathology. 2019;39(3):207-11.

[92] Margeta M, Connolly AM, Winder TL, Pestronk A, Moore SA. Cardiac pathology exceeds skeletal muscle pathology in two cases of limb-girdle muscular dystrophy type 2I. Muscle and Nerve. 2009;40(5):883-9.

[93] Martinez-Thompson JM, Moore SA, Liewluck T. A novel CAPN3 mutation in late-onset limb-girdle muscular dystrophy with early respiratory insufficiency. Journal of Clinical Neuroscience. 2018;53:229-31.

[94] Mashiah J, Harel A, Bitterman O, Sagi L, Gat A, Fellig Y, et al. Isotretinoin treatment of autosomal recessive congenital ichthyosis complicated by coexisting dysferlinopathy. Clin Exp Dermatol. 2016;41(4):390-3.

[95] Matsubara E, Tsuchiya A, Minami N, Nishino I, Pappolla MA, Shoji M, et al. A unique case of limb-girdle muscular dystrophy type 2A carrying novel compound heterozygous mutations in the human CAPN3 gene. European Journal of Neurology. 2007;14(7):819-22.

[96] McMillan HJ, Carter MT, Jacob PJ, Laffan EE, O'Connor MD, Boycott KM. Homozygous contiguous gene deletion of 13q12 causing LGMD2C and ARSACS in the same patient. Muscle and Nerve. 2009;39(3):396-9.

[97] McNally EM, Passos-Bueno MR, Bonnemann CG, Vainzof M, De Moreira ES, Lidov HGW, et al. Mild and severe muscular dystrophy caused by a single gamma-sarcoglycan mutation. American Journal of Human Genetics. 1996;59(5):1040-7.

[98] Mendell JR, Rodino-Klapac LR, Rosales XQ, Coley BD, Galloway G, Lewis S, et al. Sustained alpha-sarcoglycan gene expression after gene transfer in limb-girdle muscular dystrophy, type 2D. Annals of Neurology. 2010;68(5):629-38.

[99] Mercuri E, Brockington M, Straub V, Quijano-Roy S, Yuva Y, Herrmann R, et al. Phenotypic spectrum associated with mutations in the fukutin-related protein gene. Annals of Neurology. 2003;53(4):537-42.

[100] Meznaric-Petrusa M, Kralj E, Angelini C, Fanin M, Trinkaus D. Cardiomyopathy in a patient with limb-girdle muscular dystrophy type 2D: Pathomorphological aspects. Forensic Science International Supplement Series. 2009;1(1):58-62.

[101] Miskew Nichols B, Nikhanj A, Wang F, Freed DH, Oudit GY. Advanced Dilated Cardiomyopathy in a Patient with Hutterite Limb-Girdle Muscular Dystrophy: Use of a Left Ventricular Assist Device. Circulation: Heart Failure. 2018;11 (4) (no pagination)(e004960).

[102] Mojbafan M, Nilipour Y, Tonekaboni SH, Bagheri SD, Bagherian H, Sharifi Z, et al. A rare form of limb girdle muscular dystrophy (type 2E) seen in an Iranian family detected by autozygosity mapping. Journal of Neurogenetics. 2016;30(1):1-4.

[103] Moody S, Mancias P. Dysferlinopathy presenting as rhabdomyolysis and acute renal failure. Journal of Child Neurology. 2013;28(4):502-5.

[104] Muller T, Krasnianski M, Witthaut R, Deschauer M, Zierz S. Dilated cardiomyopathy may be an early sign of the C826A Fukutin-related protein mutation. Neuromuscular Disorders. 2005;15(5):372-6.

[105] Mustafa E, Khandaker MAH, Rashid MM, Ghose SK, Chowdhury MK. Sarcoglycanopathy - A rare case report and literature review. Journal of Medicine (Bangladesh). 2014;15(1):77-9.

[106] Nagashima T, Chuma T, Mano Y, Goto YI, Hayashi YK, Minami N, et al. Dysferlinopathy associated with rigid spine syndrome. Neuropathology. 2004;24(4):341-6.

[107] Negrao L, Geraldo A, Matos A, Rebelo O, Santos R, Marques C. The first portuguese families with limb-girdle muscular dystrophy 2L. Sinapse. 2012;12(1):39-44.

[108] Nemes A, Dezsi L, Domsik P, Kalapos A, Forster T, Vecsei L. Left ventricular deformation abnormalities in a patient with calpainopathy-a case from the three-dimensional speckle-tracking echocardiographic magyar-path study. Quantitative Imaging in Medicine and Surgery. 2017;7(6):685-90.

[109] Neto ARC, Leoni TB, De Lima FD, Martinez ARM, Nucci A, Franca MC. EMG findings in patients with limb-girdle muscular dystrophy due to heterozygous CAPN3 mutations. Clinical Neurophysiology. 2018;129 (Supplement 1):e82.

[110] Nowak KJ, Walsh P, Jacob RL, Johnsen RD, Peverall J, McNally EM, et al. Severe gamma-sarcoglycanopathy caused by a novel missense mutation and a large deletion. Neuromuscular Disorders. 2000;10(2):100-7.

[111] Oexle K, Herrmann R, Dode C, Leturcq F, Hubner C, Kaplan JC, et al. Neurosensory hearing loss in secondary adhalinopathy. Neuropediatrics. 1996;27(1):32-6.

[112] Oflazer PS, Gundesli H, Zorludemir S, Sabuncu T, Dincer P. Eosinophilic myositis in calpainopathy: Could immunosuppression of the eosinophilic myositis alter the early natural course of the dystrophic disease? Neuromuscular Disorders. 2009;19(4):261-3.

[113] Okahashi S, Ogawa G, Suzuki M, Ogata K, Nishino I, Kawai M. Asymptomatic sporadic dysferlinopathy presenting with elevation of serum creatine kinase. Typical distribution of muscle involvement shown by MRI but not by CT. Internal Medicine. 2008;47(4):305-7.

[114] Okere A, Reddy SS, Gupta S, Shinnar M. A cardiomyopathy in a patient with limb girdle muscular dystrophy type 2A. Circulation: Heart Failure. 2013;6(1):e12-3.

[115] Oliveira Santos M, Ninitas P, Conceicao I. Severe limb-girdle muscular dystrophy 2A in two young siblings from Guinea-Bissau associated with a novel null homozygous mutation in CAPN3 gene. Neuromuscular Disorders. 2018;28(12):1003-5.

[116] Paradas C, Llauger J, Diaz-Manera J, Rojas-Garcia R, De Luna N, Iturriaga C, et al. Redefining dysferlinopathy phenotypes based on clinical findings and muscle imaging studies. Neurology. 2010;75(4):316-23.

[117] Park HJ, Hong JM, Suh GI, Shin HY, Kim SM, Sunwoo IN, et al. Heterogeneous characteristics of Korean patients with dysferlinopathy. Journal of Korean Medical Science. 2012;27(4):423-9.

[118] Passos-Bueno MR, Moreira ES, Marie SK, Bashir R, Vasquez L, Love DR, et al. Main clinical features of the three mapped autosomal recessive limb-girdle muscular dystrophies and estimated proportion of each form in 13 Brazilian families. Journal of Medical Genetics. 1996;33(2):97-102.

[119] Patel NJ, Van Dyke KW, Espinoza LR. Limb-Girdle Muscular Dystrophy 2B and Miyoshi Presentations of Dysferlinopathy. American Journal of the Medical Sciences. 2017;353(5):484-91.

[120] Peddareddygari LR, Oberoi K, Baisre-DeLeon A, Grewal RP. Novel mutation in anoctamin 5 gene causing limb-girdle muscular dystrophy 2L. Journal of Clinical Neuromuscular Disease. 2018;19(4):228-31.

[121] Pena L, Kim K, Charrow J. Episodic myoglobinuria in a primary gamma-sarcoglycanopathy. Neuromuscular Disorders. 2010;20(5):337-9.

[122] Penisson-Besnier I, Richard I, Dubas F, Beckmann JS, Fardeau M. Pseudometalic variability of and phenotypic variability of calpain deficiency in two siblings. Muscle and Nerve. 1998;21(8):1078-80.

[123] Penttila S, Palmio J, Suominen T, Raheem O, Evila A, Gomez NM, et al. Eight new mutations and the expanding phenotype variability in muscular dystrophy caused by ANO5. Neurology. 2012;78(12):897-903.

[124] Peric S, Stevanovic J, Johnson K, Kosac A, Peric M, Brankovic M, et al. Phenotypic and genetic spectrum of patients with limb-girdle muscular dystrophy type 2A from Serbia. Acta myologica : myopathies and cardiomyopathies : official journal of the Mediterranean Society of Myology. 2019;38(3):163-71.

[125] Petersen JA, Kuntzer T, Fischer D, von der Hagen M, Huebner A, Kana V, et al. Dysferlinopathy in Switzerland: Clinical phenotypes and potential founder effects. BMC Neurology. 2015;15 (1) (no pagination)(182).

[126] Pimentel LHC, Alcantara RNM, Fontenele SMDA, Costa CMDC, Gondim FDAA. Limb-girdle muscular dystrophy type 2B mimicking polymyositis. Arquivos de Neuro-Psiquiatria. 2008;66(1):80-2.

[127] Pizzanelli C, Mancuso M, Galli R, Choub A, Fanin M, Nascimbeni AC, et al. Epilepsy and limb girdle muscular dystrophy type 2A: Double trouble, serendipitous finding or new phenotype? Neurological Sciences. 2006;27(2):134-6.

[128] Pollitt C, Anderson LVB, Pogue R, Davison K, Pyle A, Bushby KMD. The phenotype of calpainopathy: Diagnosis based on a multidisciplinary approach. Neuromuscular Disorders. 2001;11(3):287-96.

[129] Poppe M, Cree L, Bourke J, Eagle M, Anderson LVB, Birchall D, et al. The phenotype of limb-girdle muscular dystrophy type 2I. Neurology. 2003;60(8):1246-51.

[130] Prahm KP, Feldt-Rasmussen U, Vissing J. Human growth hormone stabilizes walking and improves strength in a patient with dominantly inherited calpainopathy. Neuromuscular Disorders. 2017;27(4):358-62.

[131] Prelle A, Comi GP, Tancredi L, Rigoletto C, Ciscato P, Fortunato F, et al. Sarcoglycan deficiency in a large Italian population of myopathic patients. Acta Neuropathologica. 1998;96(5):509-14.

[132] Prelle AS, M.; Tancredi, L.; Fagiolari, G.; Comi, G. P.; Ciscato, P.; Serafini, M.; Fortunato, F.; Zecca, C.; Gallanti, A.; Chiveri, L.; Bresolin, N.; Scarlato, G.; Moggio, M. Clinical, morphological and immunological evaluation of six patients with dysferlin deficiency. Acta Neuropathologica. 2003;105(6):537-42.

[133] Quick S, Schaefer J, Waessnig N, Schultheiss T, Reuner U, Schoen S, et al. Evaluation of heart involvement in calpainopathy (LGMD2A) using cardiovascular magnetic resonance. Muscle and Nerve. 2015;52(4):661-3.

[134] Quinlivan RM, Robb SA, Sewry C, Dubowitz V, Piccolo F, Kaplan JC. Absence of alpha-sarcoglycan and novel missense mutations in the alpha-sarcoglycan gene in a young British girl with muscular dystrophy. Developmental Medicine and Child Neurology. 1997;39(11):770-4.

[135] Rasmussen M, Scheie D, Breivik N, Mork M, Lindal S. Clinical and muscle biopsy findings in Norwegian paediatric patients with limb girdle muscular dystrophy 2I. Acta Paediatrica, International Journal of Paediatrics. 2014;103(5):553-8.

[136] Rekik S, Sakka S, Ben Romdhan S, Farhat N, Baba Amer Y, Lehkim L, et al. Novel Missense CAPN3 Mutation Responsible for Adult-Onset Limb Girdle Muscular Dystrophy with Calves Hypertrophy. Journal of Molecular Neuroscience. 2019.

[137] Renard D, Fernandez C, Bouchet-Seraphin C, Labauge P. Cortical heterotopia in LGMD2I. Neuromuscular Disorders. 2012;22(5):443-4.

[138] Ro LS, Lee-Chen GJ, Lin TC, Wu YR, Chen CM, Lin CY, et al. Phenotypic features and genetic findings in 2 Chinese families with miyoshi distal myopathy. Archives of Neurology. 2004;61(10):1594-9.

[139] Rosales XQ, Gastier-Foster JM, Lewis S, Vinod M, Thrush DL, Astbury C, et al. Novel diagnostic features of dysferlinopathies. Muscle and Nerve. 2010;42(1):14-21.

[140] Rosales XQ, Moser SJ, Tran T, McCarthy B, Dunn N, Habib P, et al. Cardiovascular magnetic resonance of cardiomyopathy in limb girdle muscular dystrophy 2B and 2I. Journal of Cardiovascular Magnetic Resonance. 2011;13 (1) (no pagination)(39).

[141] Saenz A, Ono Y, Sorimachi H, Goicoechea M, Leturcq F, Blazquez L, et al. Does the severity of the LGMD2A phenotype in compound heterozygotes depend on the combination of mutations? Muscle and Nerve. 2011;44(5):710-4.

[142] Sahai S, Khanlou N, Shieh P. A case of bilateral scapular winging. Journal of Clinical Neuromuscular Disease. 2019;20 (3):143.

[143] Sarkozy A, Deschauer M, Carlier RY, Schrank B, Seeger J, Walter MC, et al. Muscle MRI findings in limb girdle muscular dystrophy type 2L. Neuromuscular Disorders. 2012;22(SUPPL. 2):S122-S9.

[144] Sarkozy A, Hicks D, Hudson J, Laval SH, Barresi R, Hilton-Jones D, et al. ANO5 gene analysis in a large cohort of patients with anoctaminopathy: Confirmation of male prevalence and high occurrence of the common exon 5 gene mutation. Human Mutation. 2013;34(8):1111-8.

[145] Schessl J, Kress W, Schoser B. Novel ANO5 mutations causing hyper-CK-emia, limb girdle muscular weakness and miyoshi type of muscular dystrophy. Muscle and Nerve. 2012;45(5):740-2.

[146] Schneider I, Stoltenburg G, Deschauer M, Winterholler M, Hanisch F. Limb girdle muscular dystrophy type 2L presenting as necrotizing myopathy. Acta myologica : myopathies and cardiomyopathies : official journal of the Mediterranean Society of Myology / edited by the Gaetano Conte Academy for the study of striated muscle diseases. 2014;33(3):19-21.

[147] Schutz PW, Scalco RS, Barresi R, Houlden H, Parton M, Holton JL. Calpainopathy with macrophage-rich, regional inflammatory infiltrates. Neuromuscular Disorders. 2017;27(8):738-41.

[148] Sczesny-Kaiser M, Kowalewski R, Schildhauer TA, Aach M, Jansen O, Grasmucke D, et al. Treadmill training with HAL exoskeleton-A novel approach for symptomatic therapy in patients with limb-girdle muscular dystrophy-preliminary study. Frontiers in Neuroscience. 2017;11 (AUG) (no pagination)(449).

[149] Selva-O'Callaghan A, Labrador-Horrillo M, Gallardo E, Herruzo A, Grau-Junyent JM, Vilardell-Tarres M. Muscle inflammation, autoimmune Addison's disease and sarcoidosis in a patient with dysferlin deficiency. Neuromuscular Disorders. 2006;16(3):208-9.

[150] Seong MW, Cho A, Park HW, Seo SH, Lim BC, Seol D, et al. Clinical applications of next-generation sequencing-based gene panel in patients with muscular dystrophy: Korean experience. Clinical Genetics. 2016;89(4):484-8.

[151] Sharma MC, Mannan R, Singh NG, Gulati S, Kalra V, Sarkar C. Sarcoglycanopathies: a clinicopathological study of 13 cases [corrected]. Neurology India. 2004;52(4):446-9.

[152] Shouman K, Morren J, Soltanzadeh P. Phenotypic heterogeneity in limbgirdle muscular dystrophy type 2l (anoctaminopathy). Neurology Conference: 70th Annual Meeting of the American Academy of Neurology, AAN. 2018;90(15 Supplement 1).

[153] Strafella C, Campoli G, Galota RM, Caputo V, Pagliaroli G, Carboni S, et al. Limb-Girdle Muscular Dystrophies (LGMDs): The clinical application of NGS analysis, a family case report. Frontiers in Neurology. 2019;10 (JUN) (no pagination)(619).

[154] Svahn J, Streichenberger N, Benveniste O, Menassa R, Michel L, Fayolle H, et al. Significant response to immune therapies in a case of subacute necrotizing myopathy and FKRP mutations. Neuromuscular Disorders. 2015;25(11):865-8.

[155] Szymanska S, Rokicki D, Karkucinska-Wieckowska A, Szymanska-Debinska T, Ciara E, Ploski R, et al. Case report of an adolescent girl with limb-girdle muscular dystrophy type 2B - The usefulness of muscle protein immunostaining in the diagnosis of dysferlinopathies. Folia Neuropathologica. 2014;52(4):452-6.

[156] Takahashi T, Aoki M, Tateyama M, Kondo E, Mizuno T, Onodera Y, et al. Dysferlin mutations in Japanese Miyoshi myopathy: Relationship to phenotype. Neurology. 2003;60(11):1799-804.

[157] Takahashi T, Aoki M, Imai T, Yoshioka M, Konno H, Higano S, et al. A case of dysferlinopathy presenting choreic movements. Movement Disorders. 2006;21(9):1513-5.

[158] Takahashi K. Effects of prednisone on a patient with dysferlinopathy assessed by maximal voluntary isometric contraction: Alternate-day low-dose administration for a 17-year period. Case Reports in Neurology. 2019:10-6.

[159] Takano A, Bonnemann CG, Honda H, Sakai M, Feener CA, Kunkel LM, et al. Intrafamilial phenotypic variation in limb-girdle muscular dystrophy type 2C with compound heterozygous mutations. Muscle and Nerve. 2000;23(5):807-10.

[160] Tarnopolsky M, Hoffman E, Giri M, Shoffner J, Brady L. Alpha-sarcoglycanopathy presenting as exercise intolerance and rhabdomyolysis in two adults. Neuromuscular Disorders. 2015;25(12):952-4.

[161] Tasca G, Evila A, Pane M, Monforte M, Graziano A, Hackman P, et al. Isolated semitendinosus involvement in the initial stages of limb-girdle muscular dystrophy 2L. Neuromuscular Disorders. 2014;24(12):1118-9.

[162] Tasca G, Monforte M, Diaz-Manera J, Brisca G, Semplicini C, D'Amico A, et al. MRI in sarcoglycanopathies: A large international cohort study. Journal of Neurology, Neurosurgery and Psychiatry. 2018;89 (1) (no pagination)(jnnp-2017-316736).

[163] Tetreault M, Srour M, Allyson J, Thiffault I, Loisel L, Robitaille Y, et al. Founder mutation for alpha-sarcoglycan-LGMD2D in a Magdalen Islands Acadian cluster. The Canadian journal of neurological sciences. 2011;Le journal canadien des sciences neurologiques. 38(5):747-52.

[164] Todorova A, Georgieva B, Tournev I, Todorov T, Bogdanova N, Mitev V, et al. A large deletion and novel point mutations in the calpain 3 gene (CAPN3) in Bulgarian LGMD2A patients. Neurogenetics. 2007;8(3):225-9.

[165] Topaloglu H, Dincer P, Richard I, Akcoren Z, Alehan D, Ozme S, et al. Calpain-3 deficiency causes a mild muscular dystrophy in childhood. Neuropediatrics. 1997;28(4):212-6.

[166] Tsuburaya R, Suzuki T, Saiki K, Nonaka I, Sugita H, Hayashi YK, et al. Lobulated fibers in a patient with 46-year history of limb-girdle muscle weakness. Neuropathology. 2011;31(4):455-7.

[167] Ueyama H, Kumamoto T, Nagao SI, Masuda T, Horinouchi H, Fujimoto S, et al. A new dysferlin gene mutation in two Japanese families with limb-girdle muscular dystrophy 2B and Miyoshi myopathy. Neuromuscular Disorders. 2001;11(2):139-45.

[168] Umakhanova ZR, Bardakov SN, Mavlikeev MO, Chernova ON, Magomedova RM, Akhmedova PG, et al. Twenty-year clinical progression of dysferlinopathy in patients from Dagestan. Frontiers in Neurology. 2017;8 (MAR) (no pagination)(77).

[169] Vainzof M, Moreira ES, Canovas M, Anderson LVB, Pavanello RCM, Passos-Bueno MR, et al. Partial alpha-sarcoglycan deficiency with retention of the dystrophin- glycoprotein complex in a LGMD2D family. Muscle and Nerve. 2000;23(6):984-8.

[170] Van der Kooi AJ, De Visser M, Van Meegen M, Ginjaar HB, Van Essen AJ, Jennekens FGI, et al. A novel gamma-sarcoglycan mutation causing childhood onset, slowly progressive limb girdle muscular dystrophy. Neuromuscular Disorders. 1998;8(5):305-8.

[171] Van der Kooi AJ, Ten Dam L, Frankhuizen WS, Straathof CSM, Van Doorn PA, De Visser M, et al. ANO5 mutations in the Dutch limb girdle muscular dystrophy population. Neuromuscular Disorders. 2013;23(6):456-60.

[172] Viloria-Alebesque A, Bellosta-Diago E, Santos-Lasaosa S, Mauri-Llerda JA. Familial association of genetic generalised epilepsy with limb-girdle muscular dystrophy through a mutation in CAPN3. Epilepsy and Behavior Case Reports. 2019;11:122-4.

[173] Vissing CR, Preisler N, Husu E, Prahm KP, Vissing J. Aerobic training in patients with anoctamin 5 myopathy and hyperckemia. Muscle and Nerve. 2014;50(1):119-23.

[174] Von Der Hagen M, Kaindl AM, Koehler K, Mitzscherling P, Hausler HJ, Stoltenburg-Didinger G, et al. Limb girdle muscular dystrophy type 2I caused by a novel missense mutation in the FKRP gene presenting as acute virus-associated myositis in infancy. European Journal of Pediatrics. 2006;165(1):62-3.

[175] Von Guionneau A, Burford C, Stone S. Limb-girdle muscular dystrophy type 2i (LGMD2I) and dilated cardiomyopathy: Management during Pregna. International Journal of Gynecology and Obstetrics. 2018;143 (Supplement 3):583-4.

[176] Vorgerd M, Gencik M, Mortier J, Epplen JT, Malin JP, Mortier W. Isolated loss of gamma-sarcoglycan: Diagnostic implications in autosomal recessive limb-girdle muscular dystrophies. Muscle and Nerve. 2001;24(3):421-4.

[177] Wahbi K, Meune C, Hamouda EH, Stojkovic T, Laforet P, Becane HM, et al. Cardiac assessment of limb-girdle muscular dystrophy 2I patients: An echography, Holter ECG and magnetic resonance imaging study. Neuromuscular Disorders. 2008;18(8):650-5.

[178] Walsh R, Hill F, Breslin N, Connolly S, Brett FM, Charlton R, et al. Progressive dysphagia in limb-girdle muscular dystrophy type 2B. Muscle and Nerve. 2011;43(5):761-4.

[179] Walter MC, Braun C, Vorgerd M, Poppe M, Thirion C, Schmidt C, et al. Variable reduction of caveolin-3 in patients with LGMD2B/MM. Journal of Neurology. 2003;250(12):1431-8.

[180] Walter MC, Dekomien G, Schlotter-Weigel B, Reilich P, Pongratz D, Muller-Felber W, et al. Respiratory insufficiency as a presenting symptom of LGMD2D in adulthood. Acta Myologica. 2004;23(1):1-5.

[181] Walter MC, Reilich P, Thiele S, Schessl J, Schreiber H, Reiners K, et al. Treatment of dysferlinopathy with deflazacort: A double-blind, placebo-controlled clinical trial. Orphanet Journal of Rare Diseases. 2013;8 (1) (no pagination)(26).

[182] Wang CH, Liang WC, Minami N, Nishino I, Jong YJ. Limb-girdle muscular dystrophy type 2A with mutation in CAPN3: The first report in Taiwan. Pediatrics and Neonatology. 2015;56(1):62-5.

[183] Wang DN, Wang ZQ, Chen YQ, Xu GR, Lin MT, Wang N. Limb-girdle muscular dystrophy type 2I: two Chinese families and a review in Asian patients. International Journal of Neuroscience. 2018;128(3):199-207.

[184] Weiler T, Greenberg CR, Nylen E, Halliday W, Morgan K, Eggertson D, et al. Limb-girdle muscular dystrophy and Miyoshi myopathy in an aboriginal Canadian kindred map to LGMD2B and segregate with the same haplotype. American Journal of Human Genetics. 1996;59(4):872-8.

[185] Willis TA, Hollingsworth KG, Coombs A, Sveen ML, Andersen S, Stojkovic T, et al. Quantitative magnetic resonance imaging in limb-girdle muscular dystrophy 2i: A multinational cross-sectional study. PLoS ONE. 2014;9 (2) (no pagination)(e90377).

[186] Wong-Kisiel LC, Kuntz NL. Two siblings with limb-girdle muscular dystrophy type 2E responsive to deflazacort. Neuromuscular Disorders. 2010;20(2):122-4.

[187] Woudt L, Di Capua GA, Krahn M, Castiglioni C, Hughes R, Campero M, et al. Toward an objective measure of functional disability in dysferlinopathy. Muscle Nerve. 2016;53(1):49-57.

[188] Xi J, Blandin G, Lu J, Luo S, Zhu W, Beroud C, et al. Clinical heterogeneity and a high proportion of novel mutations in a chinese cohort of patients with dysferlinopathy. Neurology India. 2014;62(6):635-9.

[189] Xie Z, Xiao J, Zheng Y, Wang Z, Yuan Y. Magnetic resonance imaging findings in the muscle tissue of patients with limb girdle muscular dystrophy type 2I harboring the founder mutation c.545A>G in the FKRP gene. BioMed Research International. 2018;2018 (no pagination)(3710814).

[190] Xie Z, Hou Y, Yu M, Liu Y, Fan Y, Zhang W, et al. Clinical and genetic spectrum of sarcoglycanopathies in a large cohort of Chinese patients. Orphanet Journal of Rare Diseases. 2019;14 (1) (no pagination)(43).

[191] Xu C, Chen J, Zhang Y, Degree M, Li J. Limb-girdle muscular dystrophy type 2B misdiagnosed as polymyositis at the early stage Case report and literature review. Medicine (United States). 2018;97 (21) (no pagination)(e10539).

[192] Yilmaz A, Gdynia HJ, Mahrholdt H, Sechtem U. Cardiovascular magnetic resonance reveals similar damage to the heart of patients with Becker and limb-girdle muscular dystrophy but no cardiac symptoms. Journal of Magnetic Resonance Imaging. 2009;30(4):876-7.

[193] Ylikallio E, Auranen M, Mahjneh I, Lamminen A, Kousi M, Traskelin AL, et al. Decreased aerobic capacity in ANO5-muscular dystrophy. Journal of Neuromuscular Diseases. 2016;3(4):475-85.

[194] Yu M, Zheng Y, Jin S, Gang Q, Wang Q, Yu PL, H., et al. Mutational spectrum of Chinese LGMD patients by targeted next-generation sequencing. PLoS ONE. 2017;12 (4) (no pagination)(e0175343).

[195] Yuksel D, Dedeoglu O, Ozbay F, Talim B. A rare form of limb-girdle muscular dystrophy (LGMD type 2E) in trisomy 21. Acta Myologica. 2018;37 (2):161.
